# Aqueous Processable One-Dimensional Polypyrrole Nanostructured by Lignocellulose Nanofibril: A Conductive Interfacing Biomaterial

**DOI:** 10.1021/acs.biomac.3c00475

**Published:** 2023-07-12

**Authors:** Shujun Liang, Wenyang Xu, Liqiu Hu, Ville Yrjänä, Qingbo Wang, Emil Rosqvist, Luyao Wang, Jouko Peltonen, Jessica M. Rosenholm, Chunlin Xu, Rose-Marie Latonen, Xiaoju Wang

**Affiliations:** † Laboratory of Natural Materials Technology, Faculty of Science and Engineering, Åbo Akademi Unversity, Henrikinkatu 2, Turku FI-20500, Finland; ‡ Laboratory of Molecular Science and Engineering, Faculty of Science and Engineering, Åbo Akademi University, Henrikinkatu 2, Turku FI-20500, Finland; § Pharmaceutical Sciences Laboratory, Faculty of Science and Engineering, Åbo Akademi University, Tykistökatu 6A, Turku FI-20520, Finland

## Abstract

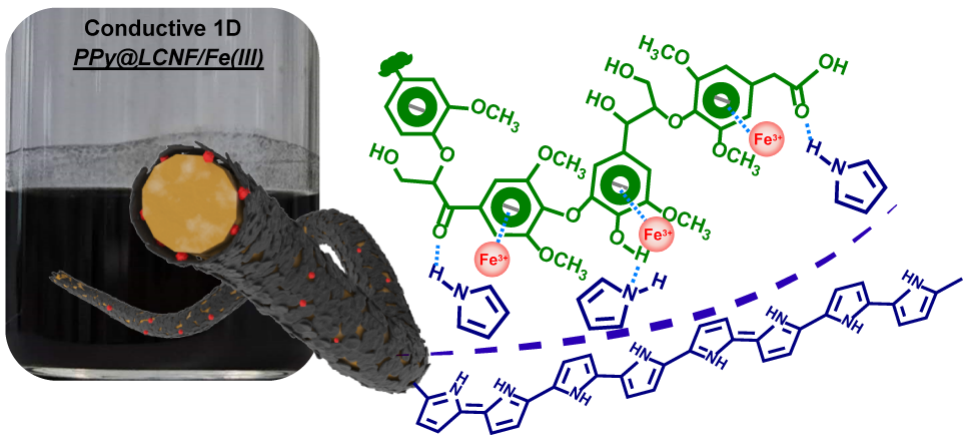

One-dimensional (1D) nanomaterials of conductive polypyrrole (PPy) are competitive biomaterials for constructing bioelectronics to interface with biological systems. Synergistic synthesis using lignocellulose nanofibrils (LCNF) as a structural template in chemical oxidation of pyrrole with Fe(III) ions facilitates surface-confined polymerization of pyrrole on the nanofibril surface within a submicrometer- and micrometer-scale fibril length. It yields a core–shell nanocomposite of PPy@LCNF, wherein the surface of each individual fibril is coated with a thin nanoscale layer of PPy. A highly positive surface charge originating from protonated PPy gives this 1D nanomaterial a durable aqueous dispersity. The fibril–fibril entanglement in the PPy@LCNFs facilely supported versatile downstream processing, e.g., spray thin-coating on glass, flexible membranes with robust mechanics, or three-dimensional cryogels. A high electrical conductivity in the magnitude of several to 12 S·cm^–1^ was confirmed for the solid-form PPy@LCNFs. The PPy@LCNFs are electroactive and show potential cycling capacity, encompassing a large capacitance. Dynamic control of the doping/undoping process by applying an electric field combines electronic and ionic conductivity through the PPy@LCNFs. The low cytotoxicity of the material is confirmed in noncontact cell culture of human dermal fibroblasts. This study underpins the promises for this nanocomposite PPy@LCNF as a smart platform nanomaterial in constructing interfacing bioelectronics.

## Introduction

In the quest toward functional biomaterials, polypyrrole (PPy) has been a favorable candidate for its ability to conduct charge coupled with the polymeric nature, long-term environmental stability, as well as for the praised biocompatibility with mammalian cells within the family of conducting polymers (CPs).^1−4^ In the fast-emerging field of biointerfaced electronics, i.e., for neural microelectrode interfacing and tissue engineering, PPys are gaining new perspectives as flexible conductive components in the fabrication of these functional devices.^5−8^ Importantly, the electrochemical responsivity of CPs offers the possibility to modulate their electrical properties with the redox processes, which are accompanied by ion flux into and out of the polymer to retain electrostatic neutrality. These properties give a unique material niche for CPs by combining electron transport (the mode of human-made electrical signals) and ionic transport (the mode of bioelectrical signals) in an organic polymer as a stimulus-responsive biomaterial suitable for bioelectronic interfaces.^9^

In chemical polymerization of pyrrole, ferric chloride (FeCl_3_) readily oxidizes the monomers to yield polymeric PPy salt, typically in a powdered form with a distinct globular morphology. The PPy salt is agreed to constitute both oxidized units containing imine-like nitrogen (N) atoms and reduced units with amine-like N atoms. The imine-like N atoms can be protonated and thus are the origins of delocalized charge carriers (bipolarons and polarons) along the polymer backbone, which account for electrical conductivity. The use of globular PPy in a functional biomaterial presents several challenges: (i) stiff and friable mechanics limiting its use as a stand-alone biomaterial; (ii) limited connectivity of the conductive network when used in a composite with nonconductive polymers; (iii) poor aqueous dispersity narrowing the processability by biofabrication; and (iv) loss of high electrical conductivity upon deprotonation when operating under physiological circumstances.^10^ In this regard, 1D nanostructures of PPy, e.g., nanofibers or nanotubes, provide superior material features compared to their granular counterpart, specifically an increased electrical conductivity, as well as a large specific surface area and interconnectivity.^11^ To facilitate the synthesis of PPy with a controlled nanomorphology, template-directed polymerization is a viable strategy to adopt in chemical polymerization.^11−13^ Well-established approaches can be exemplified by engaging the self-assembly of azo-dye or using the reactive vanadium oxide nanofibers as seed templates to guide the nanomorphology of the as-synthesized PPys.^14−16^ In particular, the underlying mechanism for the self-assembled azo dye–iron(III) complex as the seeded surface to modulate the nucleation and polymeric growth of 1D PPy nanotube is very intriguing.^17^ Nevertheless, the synthesis strategy needs an intensive effort to remove the excess dye by extraction with an organic solvent.^16,17^ Another strategy to synthesize nanostructured PPy is to incorporate a 1D nanomaterial of another kind as a structural template for in situ polymerization of pyrrole to obtain a core–shell nanocomposite with PPy as a thin shell coating while preserving the core morphology of the template. Similar strategies have been elsewhere adapted in novel syntheses of nanostructured electrode materials, resulting in a synergistic electrochemical performance.^18,19^

As strategically important biobased-nanomaterials for the 21st century, nanocelluloses, e.g., cellulose nanofibrils (CNFs) or nanocrystals (CNCs), are high-performance building blocks for constructing advanced materials and devices.^20^ CNFs produced from renewable biomass feedstock are lightweight matter with appealing characteristics such as high-aspect-ratio nanomorphology, large surface area, and high mechanical strength.^21,22^ To hybridize the electrical conductivity in a nanocomposite of CNF with electroactive materials allows the composite to inherit the high-end structural profiles, providing robust mechanics and flexibility when used for manufacturing bioelectronic interfaces or devices. More specifically, several studies have combined nanocellulose and PPy in nanocomposites.^23−25^ The presence of CNF supports the formation of freestanding membranes or monolithic aerogels featuring high electrical conductivity. Wang et al. have studied CNFs with distinct surface characteristics (neutral, anionic, or cationic) as structural templates for guiding the chemical oxidative polymerization of pyrrole.^25^ The surface properties of the CNFs have been suggested to play a decisive role in the electrical properties of the resulting nanocomposite, e.g., conductivity and capacitance. However, some challenges associated with material properties remain to be addressed. First, ensuring a continuous and homogeneous coating on nanofibrils is core to preserving the PPy nanostructure conforming to the 1D structural template. In most cases, globular PPy decorating the nanofibrils in CNFs dominates the morphology of the nanocomposite. Second, the water dispersity is poor as the doping of the polymeric backbone of PPy neutralizes the surface charge of the core CNF. This strongly impairs the processability of downstream applications, and few approaches have tackled these challenges. Tie et al. used anionic CNFs to stabilize the Pickering emulsion of cyclohexane-in-water and carried out the chemical oxidation of PPy in this reaction medium under 4 °C.^26^ In this case, the static interfacial diffusion of the pyrrole monomer caused by the solidification of the cyclohexane phase is key to ensure the formation of a PPy@CNF core–shell nanocomposite with PPy coated on individual fibrils. Wu et al. proposed the utilization of polyvinylpyrrolidone that was absorbed on the CNC surface, as a reservoir layer to enrich pyrrole monomers (via hydrogen bonding) and to promote the growth of a homogeneous PPy coating along the nanorod surface.^27^ Comparing these two aforementioned approaches, the interface- or surface-confined polymerization of pyrrole on the nanocellulose surface is the common scenario for viably modulating the synthesis of a PPy nanostructure that tightly conforms to the surface morphology of nanocellulose. To enhance the water dispersity of nanosized PPy, surfactants^28^ or polyanions, e.g., dodecylbenzenesulfonic acid,^29^ poly(hydroxyl sulfonate),^30^ and poly(styrenesulfonate),^31,32^ were used as a large-size dopant in the chemical oxidative polymerization. However, the presence of these dopants in the material system might raise concerns regarding the cytocompatibility when these PPy dispersions are used in the biological interface.^29,33^

**Scheme 1:**
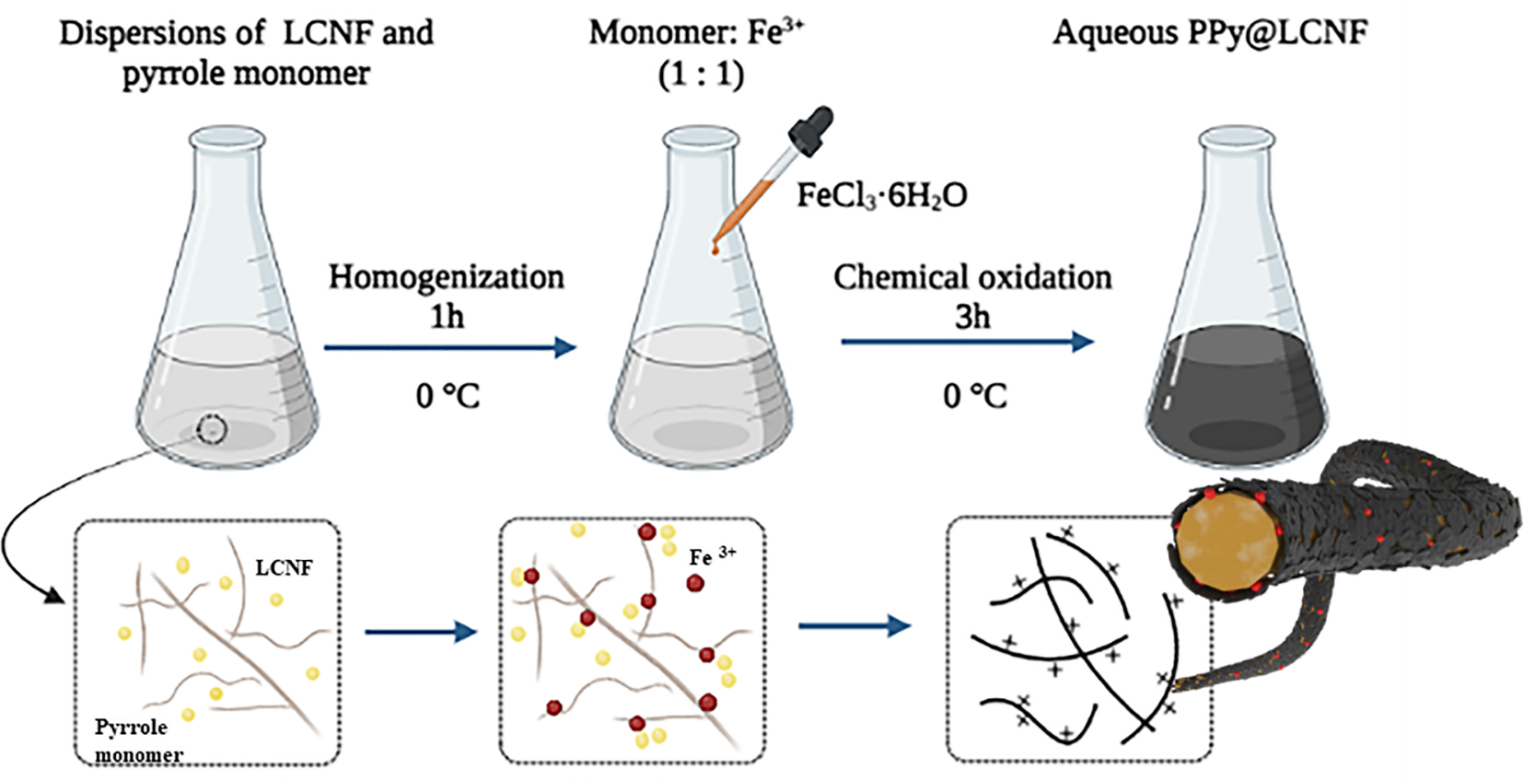
Synthesis Process of the Aqueous PPy@LCNF Dispersion

LCNFs is a class of nanomaterial in analogues to pure CNFs with respect to production method and primary nanomaterial properties.^34,35^ In the production of LCNF, the assembly of native lignin is prone to be preserved as a globular micro/nanostructure on the nanofibril surface, which gives it additional beneficial properties.^36−38^ For instance, the lignin macromolecule shows redox activity originating from the quinone/hydroquinone groups in its complex molecular structure.^39^ This redox couple potentially permits electronic interactions between lignin and the electrochemically active material, thus enabling reversible charge-transfer in an integrated system. Several reports have also confirmed that lignin effectively enhances the capacitance of pseudocapacitive polymers, including PPy.^40−42^ As another thread, the complexation of lignin with Fe(III) ions is well-documented^43^ and has been integrated in the design strategy for processing lignin-containing or lignin-derived functional materials.^44−46^ Inspired by the use of azo-dye to form a complex with Fe(III) ion as a soft template to guide the synthesis of 1D pristine PPy, we came up with the approach to use LCNF as the PPy morphology modulating hard template that we described here (Scheme 1). As lignin is preserved on the fibril surface, we hypothesize that it acts as immobilization domains for Fe(III) ions and consequently confines the polymerization of pyrrole by providing nucleation sites on the fibril surface, respectively. Ultimately, this synergetic strategy results in highly dispersed PPy@LCNF with the surfaces of individual fibrils evenly coated with a thin layer of PPy, which yields a high electrical conductivity of the nanocomposite. Moreover, the stable aqueous dispersion facilitates facile processability to manufacture various solid forms, e.g., flexible membranes by vacuum filtration, thin films on glass or steel surfaces by spray coating, and porous monolithic foams through lyophilization. To emphasize the use of the nanomaterial in bioelectronics interfacing applications, the cytocompatibility of PPy@LCNF was also evaluated in the cell culture of normal human dermal fibroblasts (NHDFs).

## Experimental Section

### Materials

LCNF and TEMPO-oxidized CNF (TCNF) are prepared in the laboratory, and the production protocols are elaborated in the Supporting Information. Pyrrole monomers (reagent grade, 98%), FeCl_3_·6H_2_O, and ammonium persulfate (APS) were purchased from Sigma-Aldrich. NHDFs (cryopreserved, C-12302) and all of the cell culture reagents were purchased from PromoCell and Gibco, respectively.

#### Material Synthesis

##### Aqueous Dispersions of Nanocomposite PPy@CNFs

Nanofibers (LCNF or T-CNF, 0.3–0.5 wt %) were first diluted with Milli-Q water (v:v, 1:1) in a double-jacket reactor and stirred for 15 min under 0 °C. The freshly distilled pyrrole monomer was added to the nanofiber suspension and rigorously mixed for 1 h to homogenize the dispersion. The molar ratio between pyrrole and the free hydroxyl groups in nanofibers was adjusted to 8:1, 12:1, and 15:1. It was assumed that the content of free hydroxyl groups is 0.0037 mol per gram dry nanofibrils, according to the previous report.^47^ The oxidant FeCl_3_·6H_2_O or APS was dissolved in a constant 4 mL of Milli-Q water and added dropwise within 15 min to initialize the in situ polymerization. The feeding molar ratio of monomer:oxidant was kept as 1:1 as the feeding ratio of pyrrole/LCNF increased across the synthesis series. The reaction was terminated after 3 h. The obtained nanocomposites were further dialyzed against Milli-Q water to remove free ions until the conductivity of dialysis water reached similar values to that of fresh Milli-Q water (∼1.5 μS cm^–1^). The purified products were further redispersed with an ultrasonic probe (Qsonic, VWR) for 1 h with pulse on–off/5 s–5 s under ice bath at an amplitude of 80%. The obtained PPy@LCNFs and PPy@TCNFs were stored at 4 °C for further use.

#### Material Processing to Solid Forms of PPy@LCNFs

##### Spray Coating

The PPy@LCNFs dispersion (0.4 wt %) was manually sprayed on the substrates (microscopic glass slides, indium tin oxide (ITO) glass, or well-polished stainless steel) using a standard airbrush pen. Spray coating was performed on a heating plate (IKAETS-D5) with a temperature-controlled program, maintaining the temperature in the range of 100–150 °C for accelerating the evaporation of water. The thickness of the resulting membranes on the glass substrates (20 × 20 mm^2^) was approximately 2 μm, as measured by atomic force microscopy (AFM).

##### Vacuum-Filtered Membrane

The PPy@LCNFs dispersion with 90 mg dry content was diluted to 0.2 wt % with Milli-Q water and vacuum-filtered through a 0.45 μm mixed cellulose ester filter (⌀ = 50 mm, Cytiva). The obtained wet membrane was air-dried under 5 kg load at room temperature. The thickness of the membrane was measured 5 times at several points with a Lorenz Wetter paper thickness meter (L&M micrometer SE250, Sweden).

##### 3D Cryogel Foam

The as-synthesized dispersion of 12PPy@LCNF (0.2 wt %, 2 mL) was frozen in liquid nitrogen and freeze-dried in a Martin CHRIST freeze-dryer (Alpha 1–4 LD Plus) to produce the 3D spongy objects.

#### Material Characterizations on Dispersion and Solid Forms of PPy@LCNFs

##### Dynamic Light Scattering (DLS) and Transmission Electron Microscopic (TEM) Analysis

All of the dispersions of PPy@CNFs were diluted to 0.01 wt % for the DLS and TEM analysis. The DLS measurement was conducted by utilizing a helium–neon laser with a wavelength of 632.8 nm and a scattering angle of 173°. The refractive index (RI) and viscosity of the Milli-Q dispersant were specified to be at 1.324 and 0.887 × 10^–3^ Pa s^–1^, correspondingly. The samples were characterized by RI and absorption values of 1.595 and 0.200, respectively. For TEM, about 5 μL of the sample was sedimented on a copper grid coated with carbon film (200 mesh, Ted Pella Inc. USA) and then incubated at room temperature for 3 min, and the excess liquid was drained with filter paper. The processed samples were loaded onto a JEM-1400 PLUS TEM microscope (JEOL Ltd., Japan) and evaluated in bright field mode with an accelerating voltage of 80 kV. Only the LCNF sample was stained.

##### Elemental Analysis

The organic C, H, and N of freeze-dried LCNF and PPy@LCNFs samples were determined in weight percentage content by an Organic Elemental Analyzer (Thermal Scientific). The O content was calculated by subtracting the sum of the contents of the above elements from 100%. The content of PPy in the composites was calculated by^33^
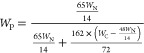
where *W*_P_: Weight percentage of PPy (%) in the composites; *W*_N_: Nitrogen content (%) from elemental analysis; *W*_C_: Carbon content (%) was from elemental analysis.

##### Scanning Electron Microscope (SEM)

The surface morphology and elemental analysis of the LCNF and PPy@LCNFs were characterized with an SEM-EDXA instrument (EDXA, LEO Gemini 1530 with a Thermo Scientific UltraDry Silicon Drift Detector, X-ray detector by Thermo Scientific). The samples were used in imaging without any additional conductive coating, and the acceleration voltage was set to 15 kV in EDXA for the sample analysis.

##### Rheological Measurement

The flow curves of LCNF and PPy@LCNFs suspensions (0.5 wt %) were measured using an MCR rheometer (Anton Paar MCR 702 Multidrive) equipped with a parallel plate geometry (PP25) with a gap distance of 0.5 mm. The shear viscosity was registered with a shear rate 0.1–1, 000 s^–1^ at 25 °C. The testing samples were presheared (100 s^–1^) for 20 s and rested for 60 s before the measurement. All measurements were carried out in triplicate.

##### Water Contact Angle (WCA)

For solid forms of LCNF and PPy@LCNFs as either vacuum-filtered membranes or spray coating onto microscopic glass slides, the WCA was measured by static contact angle measurement (KSV CAM200, KSV Instruments Ltd., Finland) with the sessile drop method. A 4 μL drop of Milli-Q water was dispensed on the membrane or coated surface at ambient temperature. The contact angle was reported at 45 s after the liquid drop landed on the surface, and measurements were carried out in triplicate.

##### Four-Probe Conductivity

The electrical conductivity of solid forms of PPy@CNFs either as vacuum-filtered membranes or spray coatings on microscopic glass slide was measured by the four-probe method using a Keithley 2400 SourceMeter with 1.82 mm tip spacing under ambient conditions (RH% = 45.9 and *T* = 22.0 °C). The designated current was applied to the sample until it was stable and reproducible, and the obtained voltages were recorded. The measurements were repeated 3 times at different points on the sample surface. The direct current (DC) conductivity was calculated followed by a correction factor with finite size electrodes in S m^–1^.^48^

##### Conductive Atomic Force Microscopy (C-AFM)

The samples were obtained by spraying PPy@LCNFs (0.4 wt %, 3 mL) on ITO substrates (7 × 7 mm^2^, thickness ∼800 nm) at 120–150 °C with a standard airbrush gun. The C-AFM was performed with a MultiMode 8 AFM instrument equipped with a Nanoscope V controller (Bruker, Santa Barbara, CA). Images of 2 × 2 μm^2^ size (resolution 512 × 512 pixels) were obtained using antimony-doped silicon cantilevers (SCM-PICV2, Bruker) with Pt–Ir coating at a 1 Hz scan speed, 100 nA V^–1^ current sensitivity, and 1 V DC current bias. The resulting images were analyzed by MountainsSPIP image analysis software with current scale cutoff values ranging from 0 to 100 nA.

##### Attenuated Total Reflectance Fourier Transform Infrared Spectroscopy (ATR-FTIR)

The internal resistance (IR) absorbance of the freeze-dried pristine LCNF and PPy@LCNFs samples were measured with an ATR-FITR spectroscope (Nicolet IS50, ThermoFisher Scientific). The spectral range was 4000–400 cm^–1^, the resolution was 4 cm^–1^, and the cumulative scans were 64.

##### X-ray Photoelectron Spectroscopy (XPS)

XPS was performed on a Nexsa XPS instrument (ThermoFisher Scientific, USA) by using monochromated Al Kα X-ray sources. All the XPS samples were subjected to an ion beam etch pretreatment (500 clusters, 8, 000 eV energy, 20 s). The XPS data was evaluated with Avantage software 5.9922 using the following parameters: (70%) Gaussian–(30%) Lorentzian Product functions were used to approximate the line shape; C1s signal at 284.8 eV was used as the internal standard for calibration of the binding energy; full width at half-maximum was constrained during the deconvolution of all core-level regions.

##### Raman Spectroscopy

Raman spectra of PPy@LCNFs as vacuum-filtered membranes were recorded with a Renishaw Ramascope imaging microscope. The spectra were recorded in the wavenumber region from 2000 to 400 cm^–1^ using an Ar ion laser with an excitation wavelength of 514 nm and a laser power of 20 mW, and with a He–Ne laser with the wavelength of 784 nm and a power of 28 mW. The spectrometer was calibrated against a silicon standard (520 cm^–1^).

#### Electrochemical Characterizations

##### Three-Electrode System Analysis

Cyclic voltammetry (CV), galvanostatic charge–discharge (GCD), and electrochemical impedance spectroscopy (EIS) were performed with an electrochemical station (reference 620 potentiostat/galvanostat/ZRA by Gamry Instruments, Inc.) equipped with a three-electrode cell configuration in 0.1 M KCl electrolyte. All the results were recorded and analyzed with the Framework and Echem Analyst, respectively. A glassy carbon (GC) disc electrode (⌀ = 3 mm, polished with 0.3 and 0.05 μm alumina slurry, sequentially) cast with a layer of PPy@LCNFs was used as the working electrode (WE). A total volume of 5 μL of the PPy@LCNFs suspension was drop-cast on the electrode surface and air-dried at room temperature. The reference electrode (RE) and the counter electrode (CE) used during CV and GCD measurements were a Ag/AgCl/3 M KCl and a platinum wire, respectively. 25-potential-cycles between −0.6 and 0.4 V versus Ag/AgCl/3 M KCl were carried out before CV, GCD, and EIS to activate the PPy@LCNFs on the electrode before starting the actual measurement. The scan rates used during the CV experiment were 0.005, 0.01, 0.025, 0.05, and 0.1 V s^–1^. A constant current density of 5 A g^–1^ was applied on the WE during the GCD experiment, and the potential cycling stability experiment was performed with 1200 cycles. The specific capacitance (*C*_sp_) from the GCD experiment was obtained by the following equation:^27^
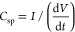
where *C*_sp_ = specific capacitance; *I* = charge current density, 5A g^–1^; and d*V*/d*t* = slope of the GCD discharge curve.

The charge capacitance (*C*_g_) can be calculated from CVs by the following equation:
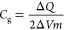
where Δ*Q* = the integrated charge from the entire voltage range; Δ*V* = the voltage window, and *m* = the mass of the PPy@LCNF films on the electrode.

The WE and RE in the EIS experiment were the same as in the GCD and CV experiments, while the CE was changed to a GC rod. The applied DC potential was 0 V vs the open circuit voltage (OCV). The AC voltage amplitude was 0.01 V in the frequency range from 1 to 20,000 Hz.

##### In Situ UV–vis Spectra

The nanocomposite PPy@LCNFs were spray coated on ITO glass (7 × 13 mm) substrates and used as WEs in the in situ UV–vis experiments. An Ag wire coated with AgCl was used as a pseudo-RE and a Pt wire was used as a CE in a customized three-electrode setup in a quartz cuvette with a path length of 10 mm. The PPy@LCNFs were doped/undoped in 0.1 M KCl electrolyte solution by stepwise applying voltage from −0.5 to 0.5 V with a potential interval of 0.1 V, while recording the spectra in situ. The spectra were recorded over the range from 250 to 1300 nm with a data collection interval of 2 nm with a High Performance PerkinElmer Lambda 1050S UV/vis/NIR Spectrometer. The detector was changed at 860 nm.

#### Cytotoxicity Analysis

The potential cytotoxicity of extract from 12PPy@LCNF membranes was assessed with the NHDFs line by Cell Counting Kit-8 (CCK-8 assay) and Live/Dead assay. Sterile membranes were immersed in culture medium in an incubator for 24 h (ratio of membrane to medium 1.25 cm^2^/mL. Condition medium with 12PPy@LCNF for 24 h (CDM24) and membranes (treated membrane) were collected and stored at +4 °C. The NHDFs were seeded into treated 96-well plates (1000 cells/well, Greiner CELLSTAR). The cells were incubated overnight in an incubator in 100 μL of fresh completed medium, ensuring that the cells were attached to the bottom of the plates. The medium was then discarded and replaced with collected CDM24 and culture dishes for the experimental and control groups, respectively, incubated in the incubator for 7 consecutive days. The CCK-8 assay and the Live/Dead assay (Calcein-AM/EthD-III) were performed on days 1, 3, 5, and 7. The detailed protocols of CCK-8 and the Live/Dead assays are shown in Supporting Information.

The cytotoxicity of the 12PPy@LCNF membranes was assessed with the NHDFs cell line by the Live/Dead assay. The NHDFs cells were seeded into 24-well plates (4000 cells/well, Greiner CELLSTAR) in 600 μL of medium and incubated overnight in an incubator, to make sure that the cells were attached to the bottom of the well. The culture medium was replaced every other day, and the insert (Falcon) with treated membrane was put into the well. The distance between the membrane and the bottom of the well was 0.8 mm. The control group was only with the insert. Cells were incubated for 5 days, and the culture medium was replaced every second day. The Live/Dead assay was performed as previously mentioned on days 1, 3, and 5.

## Results and Discussion

### Green Synthesis of Aqueous-Dispersed 1D PPy Nanostructured by LCNF

**Figure 1:**
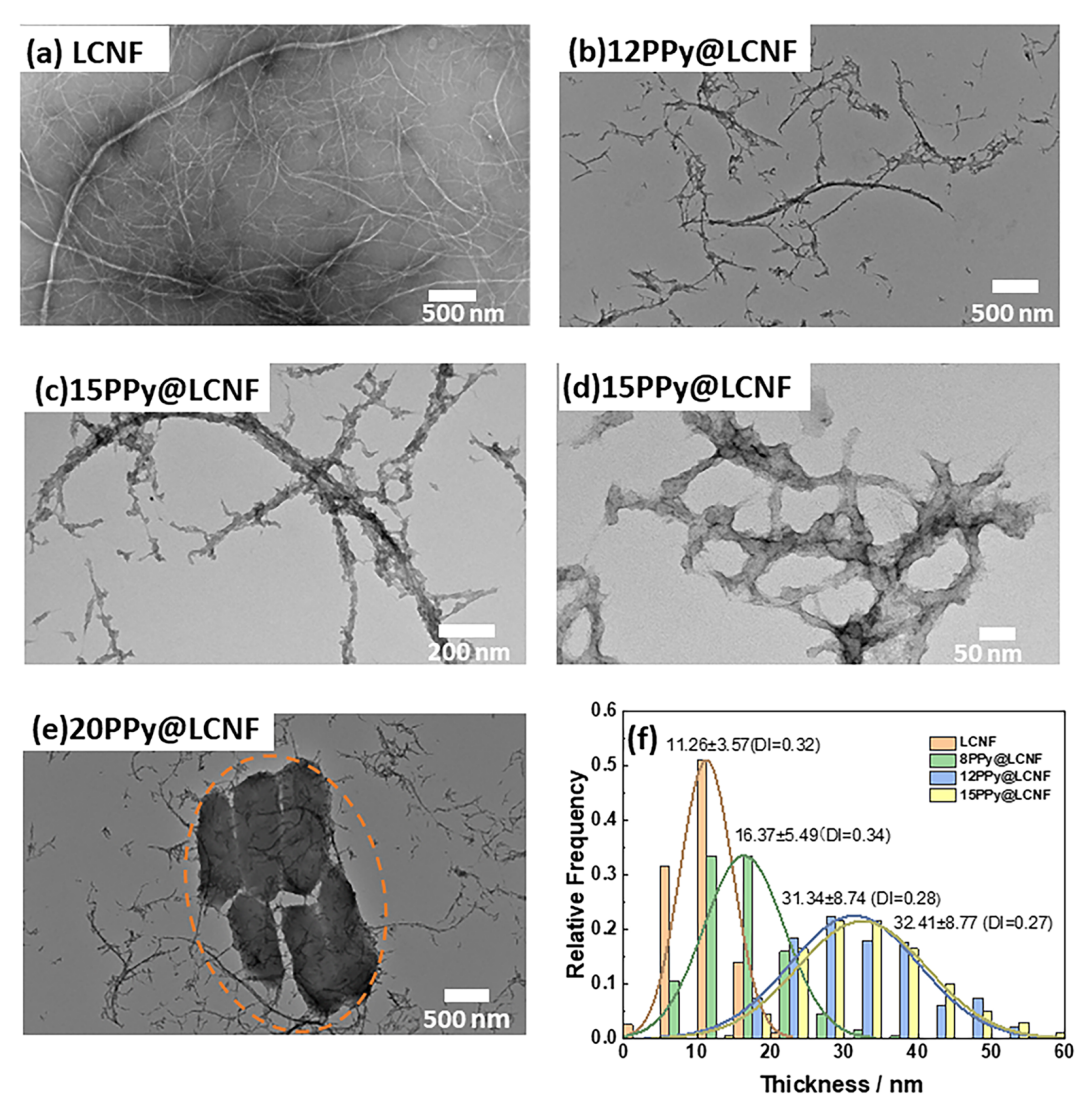
(a) TEM image of LCNF after grinding and homogenization, with magnification of 10,000×. (b) TEM image of 12PPy@LCNF with magnification of 10,000×. (c,d) TEM image of 15PPy@LCNF with magnification of 30,000×, 80,000×, respectively. (e) Bulky PPy aggregates (orange circle) in 20PPy@LCNF in 8000×. (f) Thickness distribution of LCNF and PPy@LCNFs. Note: *x* ± *y* in (f), *x* is the mean thickness (nm), while *y* is the standard deviation value (nm) of *x*. DI: Dispersity Index.

LCNFs were used as a structurally guiding 1D nanomaterial in the chemical oxidative polymerization of pyrrole with Fe(III) ions as the oxidant. The structural LCNF template has a lignin content of around 6 wt %, measured by the Klasson lignin method.^49,50^ To produce LCNFs, microfibrillated cellulose was first produced by mechanical grinding. There, the pulp fine content reached above 95.7% with fiber image analysis. The fiber product was then further defibrillated by a high-pressure homogenizer to yield LCNFs, which appeared as a translucent gel at a dry matter content of around 2 wt % (Figure S1). In TEM imaging, the fiber size distribution was confirmed to contain two subpopulations (Figure 1a); one of large-fraction nanofibrils, with a relatively thin diameter of 10 nm and short fibril length of less than one micron, and another of small-fraction microfibrils, with a relatively thick diameter up to 100 nm and long fibril length above 1 μm.

The synthesis parameters for the chemical oxidation of pyrrole were selected based on those well-argued in a study by Wu et al.^51^ Pyrrole was premixed with the LCNF suspension for 1 h to reach an equilibrium status of monomer interacting with LCNFs. A 1:1 feeding molar ratio of oxidant/monomer was kept constant with respect to the redox potential of the reaction medium, and FeCl_3_ was dropwise introduced. The reaction was carried out at 0 °C and restricted to 3 h. These reaction metrics were deployed deliberately to modulate the surface-confined polymerization of pyrrole on the fibril surface, while limiting the bulk polymerization of pyrrole, thus avoiding the formation of granular PPy. The feeding molar ratio of pyrrole monomer and LCNF (pyrrole/LCNF) was adjusted to 8:1, 12:1, 15:1, and 20:1 to optimize the synthesis of PPy@LCNF. The sample series were correspondingly denoted as 8, 12, 15, and 20PPy@LCNF. In TEM imaging, the morphology of PPy was confirmed to be an ultrathin coating tightly conforming to the core fibril surface of LCNF in three of the samples: 8, 12, and 15PPy@LCNF (Figure 1b–d and Figure S2). Apparently, PPy@LCNFs preserve the morphological features of the 1D nanofibrils in LCNFs. The thickness of the PPy coating was statistically quantified by measuring the width of random selection of 200 nano-objects within the population of the nanofibrils (length <1 μm). These results are displayed as histograms of the width of the nanofibers in each PPy@LCNF in Figure 1f. As the feeding ratio of pyrrole/LCNF increased, the average value of the thickness gradually increased from 5, 20, and 21 nm for 8, 12, and 15PPy@LCNF, respectively. However, in the 20PPy@LCNF specimen, large islands of aggregated PPy in micrometer-dimension were observed (Figure 1e). This suggests that bulk polymerization of pyrrole dominated when a high monomer concentration was deployed.

**Figure 2:**
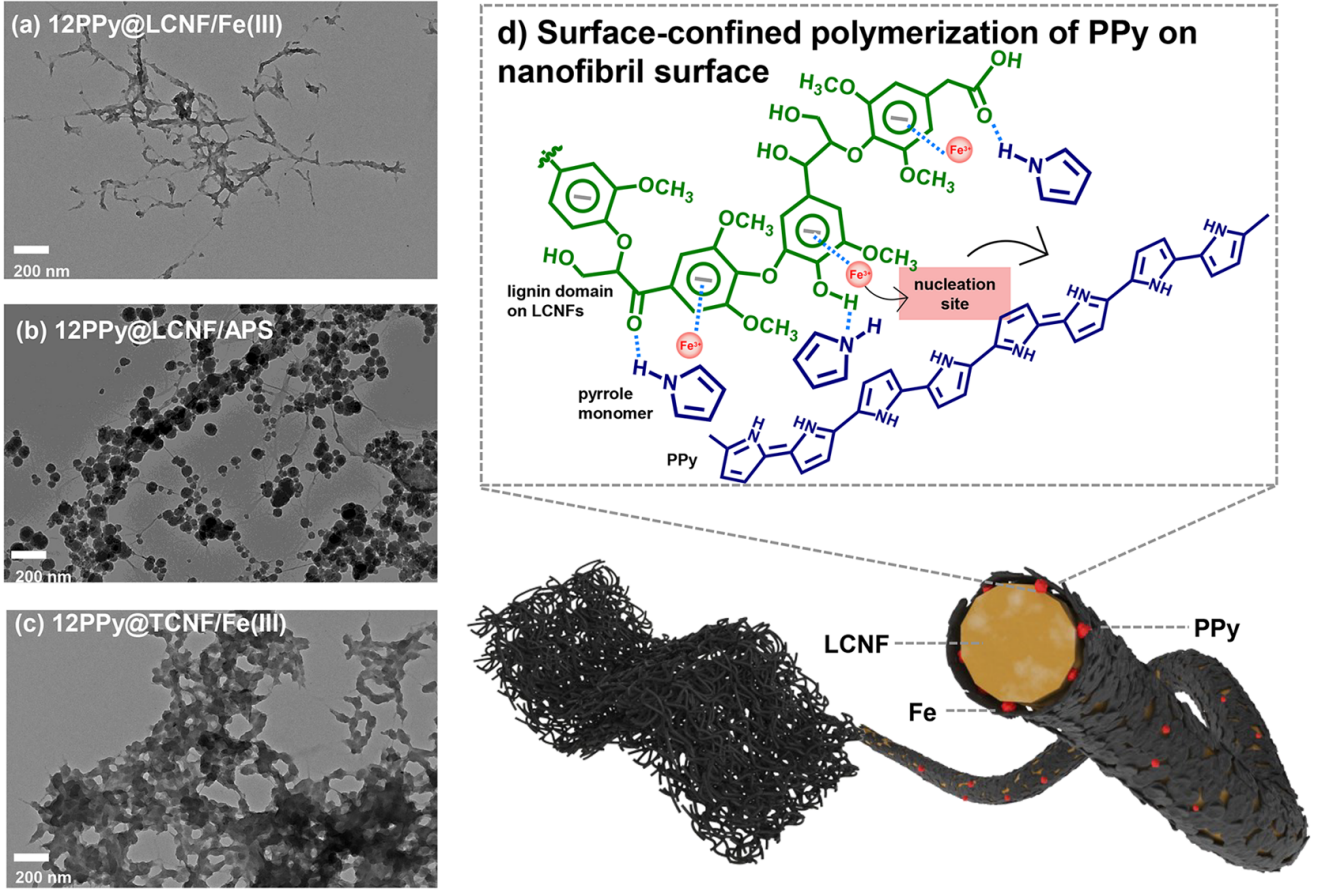
(a) TEM image of PPy synthesized on LCNF with Fe(III) ions as the oxidant. (b) TEM image of PPy synthesized on LCNF with APS as the oxidant. (c) TEM image of PPy synthesized on TCNF with Fe(III) ions as oxidant. Note: (a–c) with a scale bar of 200 nm and magnification of 20,000×. (d) Schematic illustration of the mechanism of surface-confined polymerization during the synthesis of PPy@LCNF/Fe(III).

PPy@LCNFs exhibited a positive surface charge compared to the negatively charged LCNF (Table 1). The cationic surface charge is the prime indication that the PPy in the nanocomposite is in a protonated oxidized form and is thus conductive. In addition, high values of zeta-potential (approximately +50 mV) for all three nanocomposites signify that the surface coverage of PPy is continuous on LCNF, i.e., that the LCNF fibril is extensively covered with PPy, thereby shielding the carboxylic groups and phenolic hydroxyl groups. This observation correlates well with what has been reported for the core–shell nanocomposite of PPy@CNC.^27^ To better elucidate the mechanism in the synthesis of PPy@LCNF, we also carried out the chemical oxidative polymerization under alternative conditions, either using APS as the oxidant instead of Fe(III) ions or using TCNF as the structural template instead of LCNF. When APS was used, the as-synthesized PPy was displayed as granular nanoparticles that decorated along the LCNF fibrils (Figure 2b). Unsurprisingly, the zeta-potential of 12PPy@LCNF/APS was determined as −4.5 ± 0.5 mV (Table 1). This resulted in precipitates of PPy rather than a stable dispersion (Figure S1). In polymerization with TCNF, the PPy was displayed as granular PPy nanoparticles but aggregated together with shorter TCNF fibrils (Figure 2c). 12PPy@TCNF/Fe(III) displayed a zeta-potential of −15.6 ± 0.3 mV. It remains as a short-term suspension with limited dispersity, but phase separation became visible after shelf storage for 1 week (Table 1 and Figure S1).

**Table 1:** Zeta Potential (in Milli-Q Water) and Aqueous Dispersity of Nanocelluloses and PPy@CNFs as well as PPy Morphology in the Synthesized Products (PPy@LCNFs or PPy@TCNF) with Fe(III) Ions or APS Oxidants

	Zeta potential (mV)	Aqueous dispersity^a^	PPy morphology
LCNF	–38.5 ± 0.8	Hydrogel	-
TCNF	–26.7 ± 1.2	Hydrogel	-
Fe (III) Ions As the Oxidant			
8PPy@LCNF/Fe(III)	47.7 ± 0.5	Suspension^+^	1D nanostructure; conforming to LCNF
12PPy@LCNF/Fe(III)	51.3 ± 0.8	Suspension^+^	1D nanostructure; conforming to LCNF
15PPy@LCNF/Fe(III)	50.9 ± 0.8	Suspension^+^	1D nanostructure; conforming to LCNF
20PPy@LCNF/Fe(III)	44.1 ± 0.9	Suspension^+^	Conforming to LCNF but also visible PPy aggregates
12PPy@TCNF/Fe(III)	–15.6 ± 0.3	Suspension^–^	Granular PPy aggregated with short TCNF fibrils
APS as the Oxidant			
12PPy@LCNF/APS	–4.5 ± 0.5	Aggregation	Granular PPy nanoparticles decorated along the LCNF fibril

a ^+^ acceptable dispersity over shelf-storage; ^–^ poor dispersity over shelf-storage.

By comparing with the nanocomposites that were obtained with different synthesis conditions, we propose a surface-confined polymerization mechanism for the configuration of an ultrathin PPy coating tightly conforming to the core surface of LCNF. As schematically illustrated in Figure 2d, lignin existing on the fibril surface in LCNF functions as a “molecule tank” enriching the pyrrole monomers in its vicinity via hydrogen bonding between the phenolic −O–H in lignin and the −N–H groups in pyrrole. Once Fe(III) ions are added dropwise, they are rapidly chelated and immobilized by the aromatic units in lignin. Subsequently, the polymerization of pyrrole is initiated by reacting with the lignin-Fe(III) ion complex as the nucleation site for further chain growth. Rationally, it can be deduced that π–π stacking and hydrophobic interaction favor the growth of PPy on the lignin-rich domains along the LCNF surface. Overall, this fine-tuned synergy of the lignin-Fe(III) ion complex results in the core–shell nanocomposite of PPy@LCNF. This route of manufacture highlights the “green-ness” of aqueous-dispersed PPy nanostructured by LCNFs, being synthesized without surfactants or dyes.

### Versatile Processing of Aqueous-Dispersed 1D PPy@LCNF as Conductive Surface or Monolith

**Figure 3:**
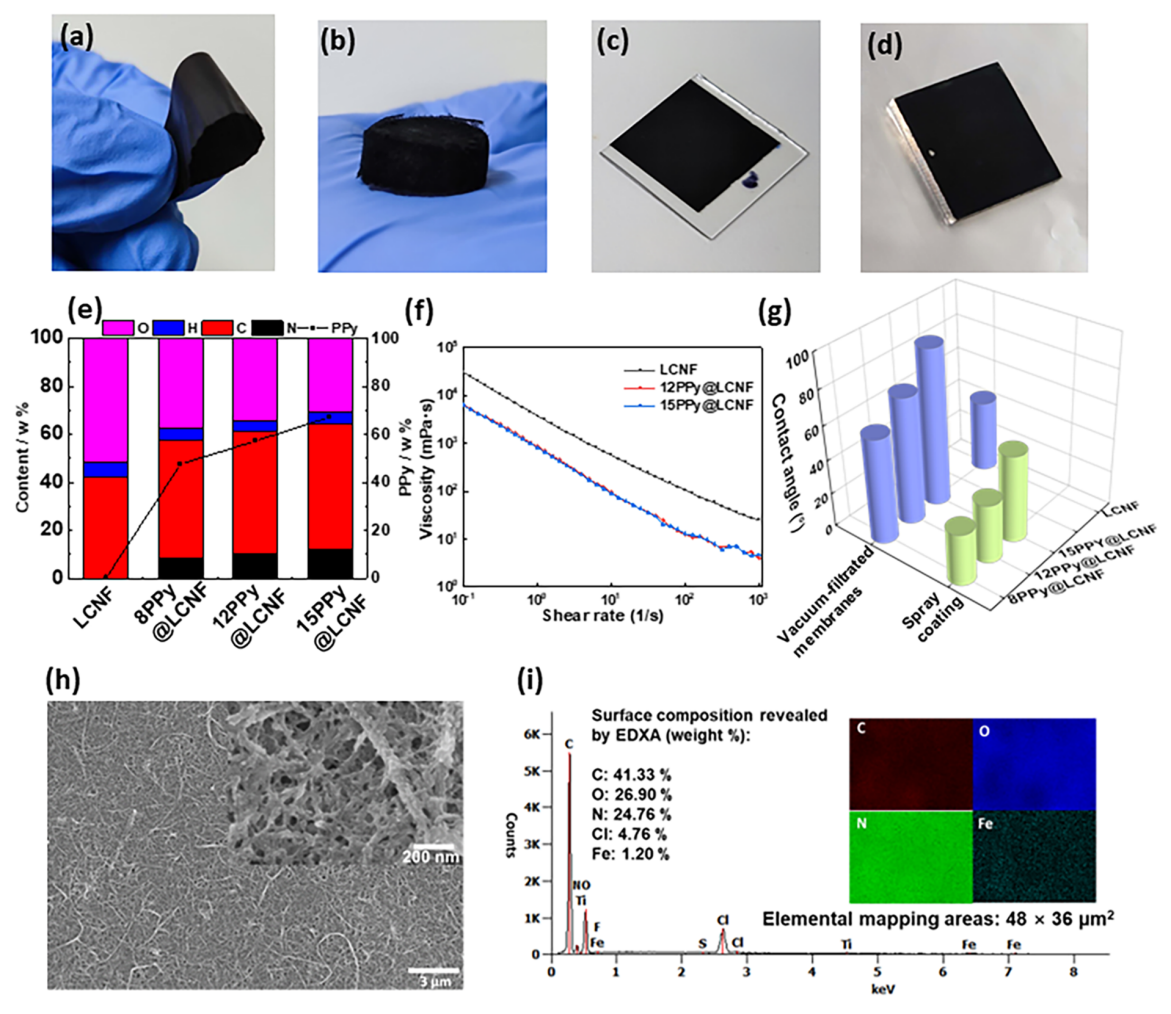
(a) Flexible PPy@LCNF membrane prepared via vacuum filtration. (b) 3D cryogel foam of PPy@LCNFs prepared via freeze-drying. (c,d) Thin coatings (less than 1 μm) via spray coating onto glass and stainless steel, respectively. (e) Elemental content of C, H, O, N, and PPy content in LCNF and PPy@LCNFs. (f) Flow curve of LCNF and PPy@LCNFs (0.5 wt %) as a function of the shear rate. (g) WCA of LCNF and PPy@LCNF solid forms fabricated via vacuum filtration or spray coating (glass surface). (h) SEM images of the 12 PPy@LCNF prepared via spray coating; the inset in (h) is the zoomed-in image. (i) EDX analysis of 12 PPy@LCNF on glass prepared via spray coating.

The aqueous dispersions of PPy@LCNF nano- and microfibrils exhibited satisfactory long-term colloidal stability during shelf storage up to one month (Figure S1), bestowed by the high positive surface charge of protonated PPy. Since the PPy nanocoating tightly conforms to the morphology of the structural template, the fibril geometry was well preserved in the nanocomposite, and the elementary PPy@LCNF was displayed as a flexible and conductive 1D nanomaterial. At a dry content of 0.5 wt %, the 12 and 15PPy@LCNF dispersions showed a shear-thinning behavior as reflected by the rheological flow curves (viscosity vs shear rate) (Figure 3f). Compared to LCNF, the viscosity of 12 and 15PPy@LCNF greatly decreased, which indicates that the surface properties of the nanocomposite differ from those of LCNF. The high-aspect-ratio nanomorphology of PPy@LCNF is beneficial to render an interconnected 3D network via intrinsic fibril entanglement in the solid forms.

**Figure 4:**
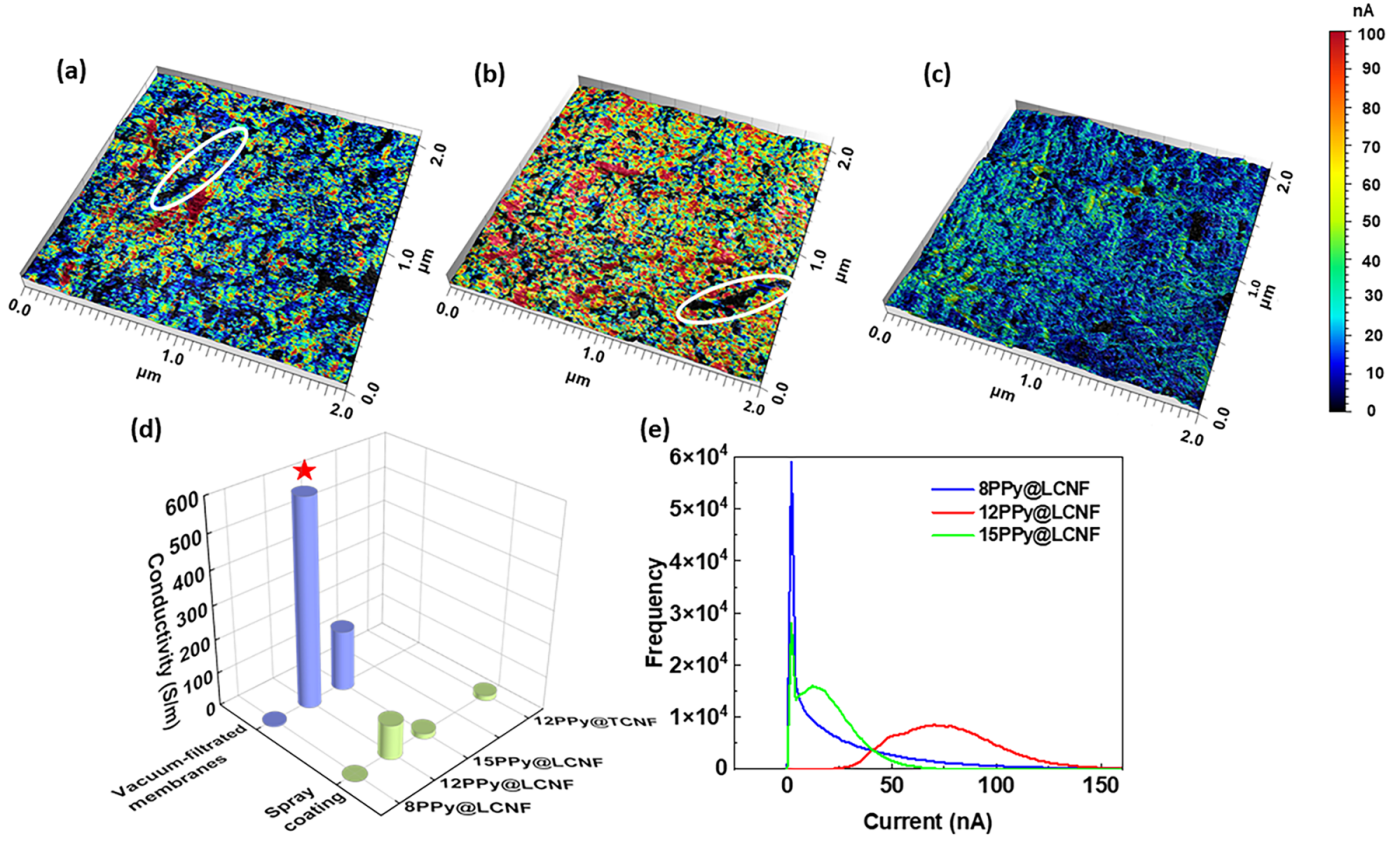
Conductivity maps obtained with C-AFM (2 × 2 μm) of (a) 8PPy@LCNF, (b) 12PPy@LCNF, and (c) 15PPy@LCNF. (d) Four-probe conductivity of PPy@LCNFs and 12PPy@TCNF via vacuum filtration or spray coating. (e) Current (nA) distribution density in the C-AFM images for PPy@LCNFs. Note: red star in (d) denotes the conductivity of 12PPy@LCNF membrane increased from 6.0 to 12.7 S cm^–1^ after the postal soaking-wash step in 0.3 M HCl.

To demonstrate the versatility of processing dry applications of PPy@LCNFs, we prepared three different solid forms: mechanically strong freestanding membranes through vacuum filtration (thickness of 20–30 μm; Figure 3a); 3D cryogel foam via lyophilization (Figure 3b); and robust thin coatings (less than 1 μm) on glass and stainless-steel surfaces (Figure 3c and d). In the C–H–N–O elemental analysis, the content of PPy was 47.6%, 57.3%, and 67.3% in 8, 12, and 15 PPy@LCNF, respectively. The PPy content in the sample series correlates well with the statistical counting of the fibril diameters (Figure 1f). Under SEM imaging (Figure 3h), a highly interconnected fibril–fibril network was prominent in the spray-coated sample, and a continuous morphology of PPy coated on the nanofibrils was further confirmed in a high-magnification image (Figure S3b and inset in Figure 3h). Elemental mapping performed on a sample area of 48 × 36 μm^2^ revealed that the N, which indicates the presence of PPy, was homogeneously distributed throughout the measuring zone (Figure 3i). Elemental Fe (1.2 wt % content) was also seen distributed throughout the sample (Figure 3i). This further supports the hypothesized mechanism of the surface-confined polymerization of pyrrole on the LCNF surface. Elemental Cl was also confirmed in the energy-dispersive X-ray analysis (EDXA) of the spray-coated specimen of 12PPy@LCNF, which is expected as the charge of the oxidized PPy is balanced with the Cl^–^ dopants in the polymeric network. The WCA of PPy@LCNF increased with PPy molar ratio of the stem dispersion from moderately hydrophilic 8PPy@LCNF (61°), to 12PPy@LCNF (74°), and further to neither hydrophilic nor hydrophobic 15PPy@LCNF (90°) in the vacuum-filtered membrane series (Figure 3g). As a control, the LCNF membrane displayed a higher hydrophilicity with a WCA of 41°. The same trend was confirmed with the sample series of sprayed thin coatings. The surface wettability strongly indicates that the surface-confined polymerization of pyrrole gradually converts the hydrophilic lignocellulose surface to a less hydrophilic PPy surface. Both the vacuum-filtered membrane and the spray-coated 15PPy@LCNF showed the highest WCA of 90° and 60°, respectively (Figure 3g). The difference in the WCA between the filtered membrane and the sprayed thin coating can be associated with surface topography. The vacuum-filtered membrane showed a more densely packed fibril–fibril network compared to the spray coatings on the glass surface, as revealed by SEM (Figure S3b). For the respective sample series, the highest DC conductivity was determined by the four-point van der Pauw method as 6 S cm^–1^ for the vacuum-filtered membrane and as 1 S cm^–1^ for the spray coating on glass (Figure 4d). This difference is mainly associated with the connectivity of the conductive network or, in other words, the compactness of the conductive fibrils in the solid forms. As the PPy content increased, a great increase in electrical conductivity was observed for 12PPy@LCNF compared with 8PPy@LCNF. Surprisingly, the electrical conductivity dropped for 15PPy@LCNF, though more PPy was obtained in the surface-confined polymerization as confirmed in composition analysis (Figure 3e). The feeding ratio of the oxidant/monomer ratio was kept at 1:1 as a statistical metric, and a higher concentration of Fe(III) ion was introduced dropwise in the case that this feeding ratio was engaged higher. It is postulated that for the 15PPy@LCNF, the surface-confined oxidant/monomer ratio at the local regime close to the fibril surface might be high, which would result in rapid polymerization, and thus less ordered packing of the macromolecules on the LCNF. Similarly, a drop in electrical conductivity of the PPy nanotubes synthesized with the self-assembly of methyl orange has been previously discussed to be associated with a less ordered structure of PPy.^52^

C-AFM was used to reveal the area distribution and density of conductive paths on nanoscale in the spray-coated samples on ITO glass, where ionic and electronic currents are passing through the material surface.^53^ 12PPy@LCNF had significantly more highly conductive path distribution (red colored area in Figure 4b) than other samples. Meanwhile, the abundance of highly conductive data points with currents above 50 nA was greater for 8PPy@LCNF than for 15PPy@LCNF (red and yellow/green areas in Figure 4a and c). Interestingly, 15PPy@LCNF had more conductive data points in the 10–40 nA region. Figure 4e shows the distribution of conductivity at different data points for the PPy@LCNFs with a 1 V DC bias, which was obtained by summarizing the frequency of all the conductive points in at least two images for each sample in different places. The high conducting points in 12PPy@LCNF are centralized in the range of 50–100 nA, indicating a more concentrated and homogeneous conducting network, while the conducting points in 8 and 15PPy@LCNF are much broader and concentrated in the lower conducting range (<40 nA). This further supports our hypothesis that a high electrical conductivity is facilitated by a comparatively low feeding ratio of pyrrole/LCNF, albeit at the sacrifice of a small fraction of the LCNF surface not forming conductive sites (white circles in Figure 4a and b). To summarize, fine-tuning the feeding ratio of pyrrole/LCNF is crucial when synthesizing PPy@LCNF with a high electrical conductivity.

The DC conductivity of the vacuum-filtered membrane of 12PPy@LCNF was on the order of magnitude of several S cm^–1^. This falls into the range reported for pelletized globular PPyCl particles prepared by chemical oxidation^54^ but lower than the high conductivity close to 100 S cm^–1^ as reported for compressed pellets of pristine PPy nanotubes that were synthesized with the self-assembled organic dye as the morphology-guiding agent.^16^ Whereas, the conductive nano- and macrofibrils in PPy@LCNF inherit strong and flexible mechanics, which can readily address the mechanical durability of PPy as a stand-alone membrane. We summarized various cellulose-based conductive platforms as reported in the literature in Table 2. The obtained electrical conductivity of the vacuum-filtered membrane of 12PPy@LCNF is higher than what was reported for composites of nanocellose/PPy or nanocellulose/reduced graphene oxide (rGO).^55,56^ In some studies, the monolith fabrication was carried out through in situ polymerization of pyrrole within the paper substrate or wood tissue, which restricted the processability.^40,42,57^ In this perspective, the facileness to process PPy@LCNFs as various solid forms is advantageous in adapting versatile scenarios in building bioelectronic interfaces and devices. It is worth noting that the protonation level of PPy strongly depends on the experimental conditions of its preparation. In our synthesis protocol, strong acid was not used, as the phenolic −OH in lignin can be protonated at a low pH, which would impede the complexation of Fe(III) ions with lignin structure and thus negatively affect the surface-confined polymerization of pyrrole on individual fibrils. This phenomenon was verified when optimizing the synthesis (Figure S4). In the case that PPy@LCNFs would be used as an elementary building block in a biomaterial system for interfacing with cultured cells under pH-neutral conditions, a highly protonated PPy would encounter deprotonation and a consequent decrease of electrical conductivity as reported elsewhere.^58,59^ Hence, we did not perform a wash step with strong acids when processing the PPy@LCNF suspension as a solid form. In fact, the DC electrical conductivity measured for the vacuum-filtered membrane of 12PPy@LCNF increased to 12 S cm^–1^ upon a soaking-wash step in 0.3 M HCl for the fabricated solid form (red star label in Figure 4d).

**Table 2:** Comparison of Electrical Conductivity of PPY@LCNF with Reported Conductive Composites of PPy with Cellulose or Wood Tissue

Composites	DC conductivity (S cm^–1^)	ref
Nanocellulose/PPy composite	1	(55)
Nanocellulose/rGO	0.7	(56)
**PPy@LCNF**	**12**	**Current work**
Monolith Fabrication on Paper Substrate or Wood Tissue		
PPy-coated paper	15	(57)
PPy-coated paper with alkaline lignin as dopant in PPy	24.8	(57)
Nanoporous cellulose gel coated with PPy nanoparticles	0.08	(33)
Wood/PPy porous composites with lignin sulfonation treatment	1.8	(42)

### Spectroscopic Characterizations of PPy@LCNF

**Figure 5:**
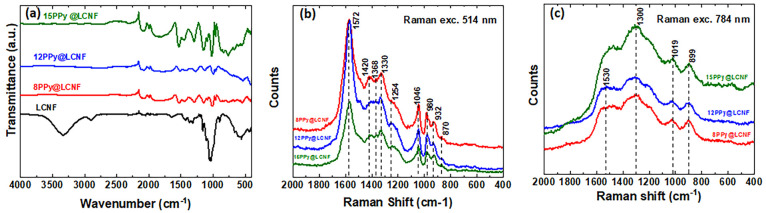
(a) ATR-FTIR spectra of the LCNF and PPy@LCNFs. Raman spectra of PPy@LCNFs at excitation wavelengths (b) 514 nm and (c) 784 nm.

To verify the chemical nature of PPy as obtained in PPy@LCNF, multiple spectroscopic characterizations were conducted. LCNF and the PPy@LCNFs were characterized by ATR-FTIR. The infrared spectra were normalized to the most pronounced peak at 1036 cm^–1^, which is ascribed to the C–O stretching vibration of polysaccharides and lignin.^60^ For LCNF (Figure 5a), characteristic peaks for lignocellulose were confirmed at 3330 cm^–1^ (aromatic and aliphatic O–H stretching vibration), at 2906 cm^–1^ (C–O stretching vibration), at 1645 cm^–1^ (O–H bending from absorbed water or conjugated C=O stretching), at 1429 cm^–1^ (C–H_2_ bending of the pyranose ring), at 1373 cm^–1^ (C–H bending), at 1161 cm^–1^ (aromatic C–H in-plane deformation), at 1036 cm^–1^ (C–O–C pyranose ring vibration), and at 897 cm^–1^ (β-glycosidic linkage between glucose units in cellulose), respectively.^61,62^ For the PPy@LCNFs, the ATR-FTIR spectra mainly present the characteristic bands of PPy, with maxima at 1535 cm^–1^ (C=C or C–C stretching vibrations in the pyrrole ring), at 1443 cm^–1^ (C–N stretching vibrations in the ring), at 1295 cm^–1^ (=C–H or C–N in-plane deformation modes), and a local absorption maximum at 1163 cm^–1^ (breathing vibrations of the pyrrole ring).^63,64^ First, ATR-FTIR data confirmed the presence of PPy in the nanocomposite. Second, the hydroxyl-related peaks were greatly influenced by the formation of PPy on LCNF. Such peaks in LCNF, as the ones with maxima at 3330 cm^–1^_,_ 2906 cm^–1^, 1645 cm^–1^, and 897 cm^–1^, were not detected in the infrared spectra of PPy@LCNFs. These distinctions indicate a high level of coverage of PPy on the LCNF and suggest that the interfacial interaction between PPy and LCNF might be associated with hydrogen bonds between the hydroxyl oxygen atom in LCNF and the nitrogen atom in PPy. This type of interaction is hypothesized as one of the mechanistic scenarios facilitating the surface-confined polymerization of PPy on LCNF.

Raman spectroscopy is an essential method to evaluate the structural bonding in CPs and is sensitive to localized structural defects associated with the existence of polarons and bipolarons in the protonated PPy.^52^ In the measurements, we used two laser excitation wavelengths, 514 and 784 nm. As the laser excitation wavelength of 514 nm is close to the minimum of electronic absorption of PPy, the neutral (reduced) form of PPy strongly resonates at this given excitation wavelength. The most dominant Raman scattering peak for PPy@LCNFs was at 1572 cm^–1^, assigned to the C=C stretching vibrations of the PPy backbone (Figure 5b). Other bands of PPy were found with maxima at 1368 and 1330 cm^–1^ (bands of ring-stretching vibrations) and 1046 cm^–1^ (C–H out-of-plane deformation vibrations), and double peaks at 980 and 932 cm^–1^ (ring-deformation vibrations of neutral PPy and ring-deformation vibrations in bipolaron units, respectively) were also detected in the spectra of 8, 12, and 15PPy@LCNF (Figure 5b). The band with a maximum at 1254 cm^–1^ (antisymmetric C–H deformation vibrations) was only prominent in the spectra of 12PPy@LCNF and 15PPy@LCNF. The energy of laser excitation wavelength at 784 nm is in resonance with the energy of delocalized polarons and bioplarons. In the Raman spectra of PPy@LCNFs recorded with a 784 nm excitation laser, the band at 1530 cm^–1^ (emerged from the C=C stretching vibrations of the PPy backbone) is observed as a broad band (Figure 5c). In addition, the bands associated with the ring-stretching vibrations are broad and centered at 1300 cm^–1^. A peak with a local maximum at 1019 cm^–1^ can be attributed to the out-of-plane C–H deformation vibrations. The band centered at 899 cm^–1^ is considered to be associated with the ring-deformation vibrations of bipolaron units in PPy, which has shifted to lower frequencies compared to the band at 932 cm^–1^ in the spectra recorded with 514 nm excitation wavelength. It has been agreed that Raman spectra of chemically prepared PPy at a given excitation wavelength are strongly dependent on the protonation degree.^65^ Under the experimental conditions of its preparation, the PPy@LCNFs are in their intermediate protonated state, which might account for the broadening of peaks under excitation at 784 nm.

**Figure 6:**
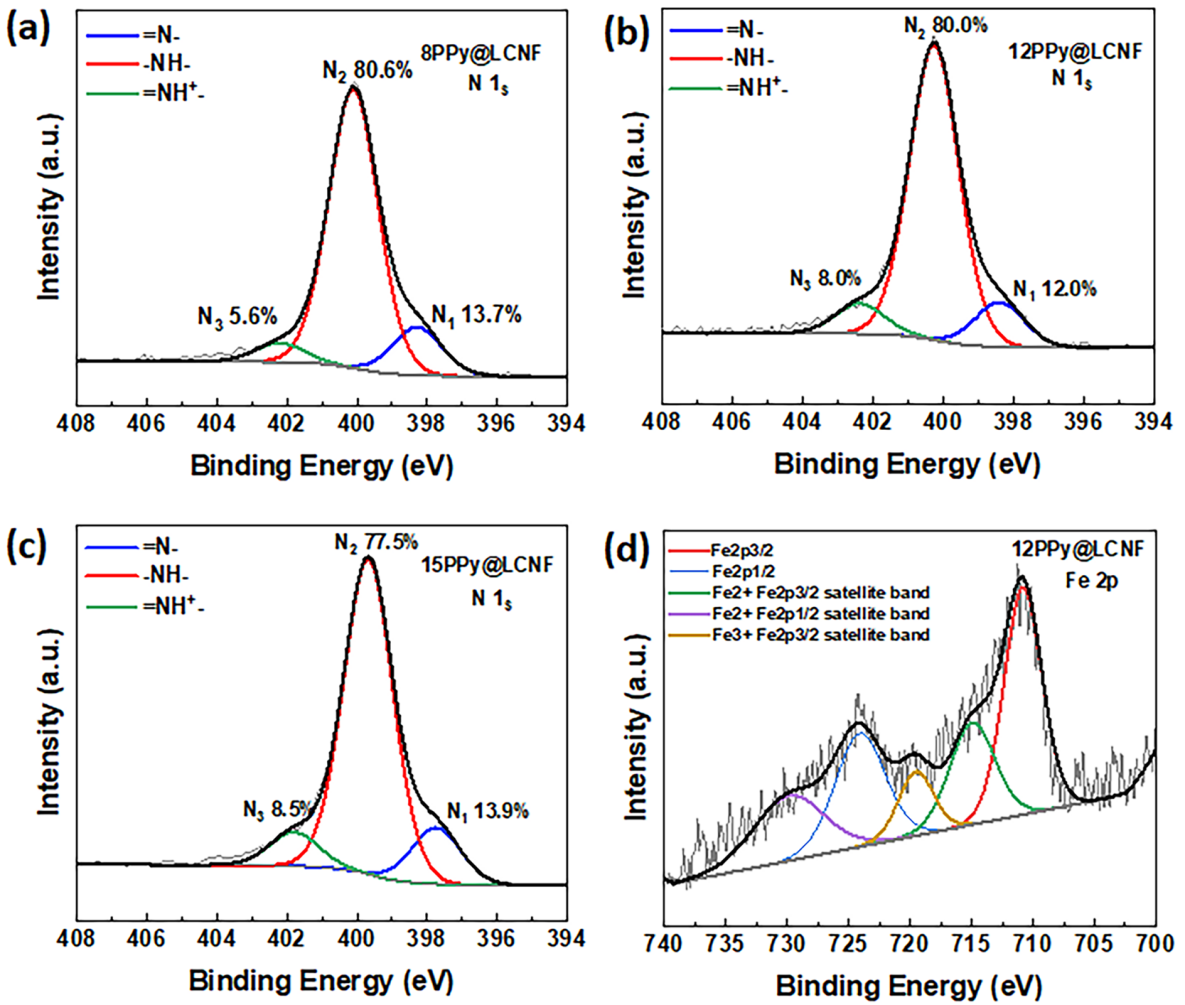
High-resolution XPS spectra over N 1s core-level regions of (a) 8PPy@LCNF, (b) 12PPy@LCNF, (c) 15PPy@LCNF, and (d) the valence of Fe for 12PPy@LCNF.

According to the XPS measurements, the O content decreased and the C, N, and Cl content increased across the samples series from 8, 12, to 15PPy@LCNF (Table S1a). This confirms that the surface of PPy@LCNF presents more protonated PPy as the feeding ratio of pyrrole/LCNF increased under the synthesis conditions. The 12PPy@LCNF exhibited the highest atomic Cl/N ratio, suggesting a higher doping level that accounts for the highest as-measured DC conductivity. Moreover, XPS data provide critical information on the electron valence of N and Fe elements present in the nanocomposite to gain more insight into the chemical status of the PPy@LCNFs. As revealed by the deconvoluted bands of N 1s core level region (Figure 6a–c), deprotonated imine (=N–, N_1_), neutral N atoms (−NH–, N_2_), and protonated imine (=NH^+^–, N_3_) species are all present. The neutral N atoms account for 77–80% of the total N atoms, which is consistent with what has been reported elsewhere for the PPy salts synthesized by chemical oxidation (Table S1b).^66^ Intermediate levels of protonation, with respect to the ratio of N_3_/N_1_, are suggested for the PPy@LCNFs obtained with the current synthesis protocol. Nevertheless, 12PPy@LCNF (0.67) and 15PPy@LCNF (0.61) gave a comparatively higher ratio of N_3_/N_1_, when compared with 8PPy@LCNF (0.41) (Table S1b). This observation is consistent with the results of the DC conductivity measured for this sample series. The deconvoluted bands of the Fe 2p core level region confirmed both iron valences of Fe^2+^ and Fe^3+^ (Figure 6d). As mentioned previously, we propose that the complexation of Fe(III) ions with the aromatic structure of lignin facilitates the surface-confined polymerization of pyrrole on the nanofibrils. We further postulate that the complexed Fe(III) ions are immobilized in the nanocomposite PPy@LCNF. Mechanistically, Fe(III) ions that are complexed with lignin to locally oxidize the pyrrole monomers would be preserved as Fe(II) ions, where they possibly are sandwiched in the nanocomposite, as the nucleation for the chain growth of PPy is expected to occur in close proximity of the lignin structure.

### Electrochemical Properties of PPy@LCNFs

**Figure 7:**
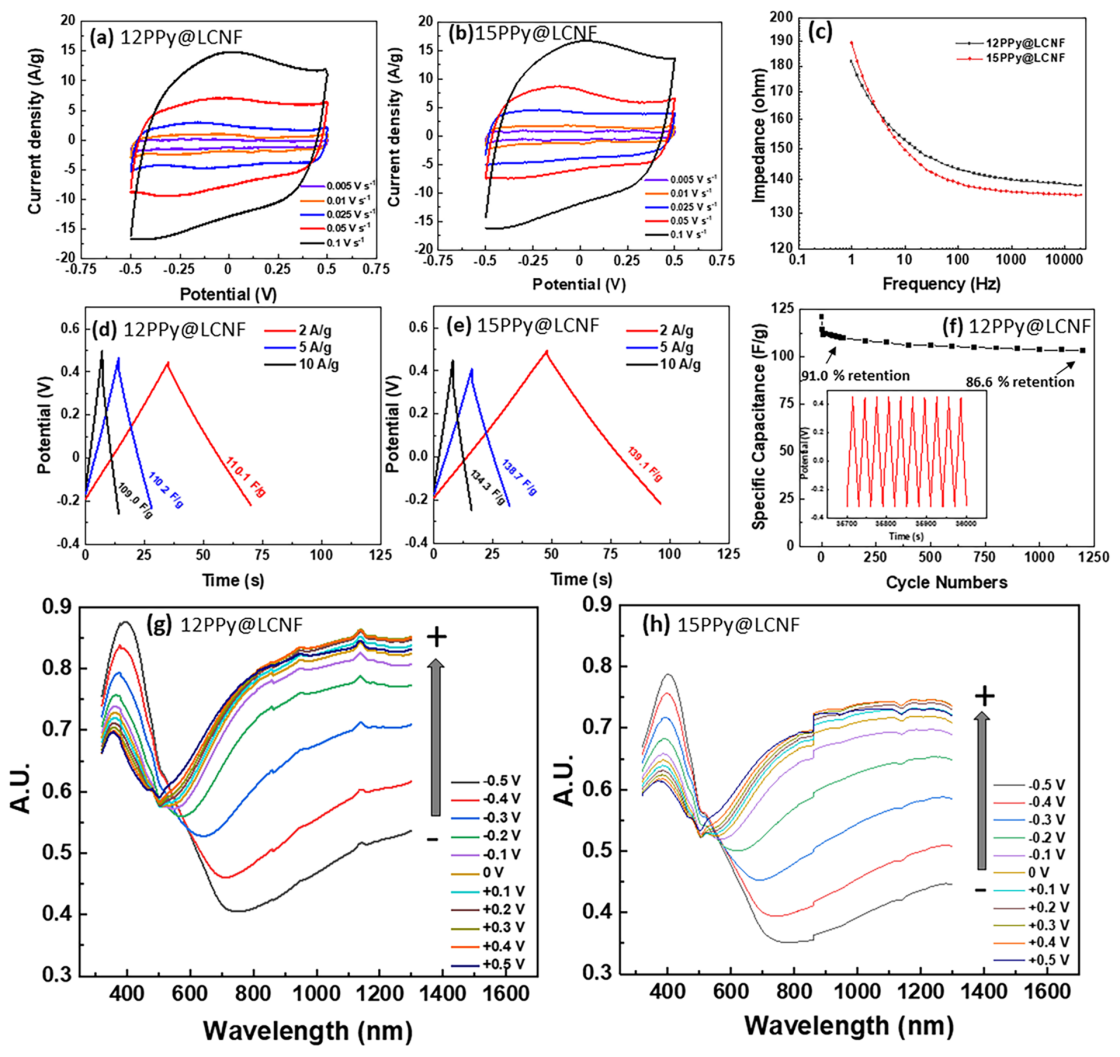
Electrochemical characterization of nanocomposite electrodes of PPy@LCNFs in 0.1 M KCl recorded in an asymmetric three-electrode electrochemical cell with Ag/AgCl (3 M KCl) as the RE. CV curves for (a) 12PPy@LCNF and (b) 15PPy@LCNF with varied scan rates. (c) Bode plots for 12 and 15PPy@LCNF as measured by EIS. Charge–discharge curves recorded at different current densities for (d) 12PPy@LCNF and (e) 15PPy@LCNF. (f) Cycling performance in terms of capacitance retention at 5 A g^–1^ for 12PPy@LCNF (the capacitance from the first cycle is set to 100%). In situ UV–vis spectra of the spray-coated nanocomposite of (g) 12PPy@LCNF and (h) 15PPy@LCNF on ITO-glass measured between −0.5 and +0.5 V in 0.1 M KCl. The potential is applied vs a pseudo-RE of Ag wire coated by AgCl.

“Standard” PPy salt is electrochemically active, and the oxidation/reduction cycle of PPy synchronizes the doping/undoping process of mobile ions in and out of the polymeric structure as an ion flux, referring to ionic conductivity of PPy. The electrochemical property of the nanocomposite PPy@LCNFs was probed through CV, GCD, and EIS in an asymmetric three-electrode setup in an aqueous electrolyte of 0.1 M KCl. The utilization of a low-concentration electrolyte is used to simulate the scenario of ion strength in physiological buffers. Without using any extra binder or conductive additive, the aqueous suspension of 12 or 15PPy@LCNF was drop cast on a well-polished GC surface, and an intact membrane of PPy@LCNF was formed, fully covering the GC electrode surface. This electrode serves as the WE in the electrochemical cell, and the robust adhesion of the PPy@LCNF nanomaterial to the GC surface guarantees good electrical contact with the current collector. The WEs of 12 and 15PPy@LCNF displayed an OCV of 0.22 and 0.21 V, respectively, versus the RE of Ag/AgCl (3 M KCl). The redox status, as reflected by these OCVs also indicates an intermediate level of protonation in the PPy@LCNFs. Both 12 and 15PPy@LCNF showed rectangular-like CV curves at scan rates of 0.005–0.1 V s^–1^ shown in Figure 7a and b, which displayed reversibility of the electrochemical redox processes within the potential window of −0.5 to 0.5 V. As suggested by the integrated surface area of the CV graphs, the interconnected nano- or microfibrils of PPy@LCNF are hypothesized to enhance the interchain charge transport and to result in enhanced pseudocapacitance. 15PPy@LCNF showed a larger value of gravimetric capacitance than 12PPy@LCNF at the same scan rate based on the calculation with respect to the mass of the total composite (PPy + LCNF). The gravimetric capacitance of 12PPy@LCNF retained 84.0% of its capacitance when the scan rate was increased from 0.005 to 0.1 V s^–1^, suggesting excellent charge transfer kinetics (Figure S5). The potential cycling stability of 12PPy@LCNF was confirmed by 200 CV cycles with a scan rate of 0.25 V s^–1^. (Figure S6). EIS provides information about the charge transfer characteristics of the film, and we used this technique to monitor the behavior of 12PPy@LCNF and 15PPy@LCNF samples over a frequency range of 1–20,000 Hz. Their Bode plots and Nyquist plots are summarized in Figure 7c and Figure S7, respectively. As seen in the Nyquist plots, the impedance response at low frequencies indicated capacitive behavior and low charge diffusion resistance for the PPy@LCNFs as a conductive membrane attached to the GC surface. In the Bode plots, the magnitude of impedance decreases with an increase in frequency. 15PPy@LCNF shows an impedance lower than that of 12PPy@LCNF, which is indicative of a larger capacitance for the former. As most neural cell communication occurs between 300 and 1000 Hz, the impedance measured is commonly examined as a biologically important frequency at 1000 Hz.^67,68^ At 1000 Hz, the measured values for PPy@LCNFs are comparable to those reported for the conductive hydrogel of poly(2-hydroxyethyl methacrylate) integrated with PPy used for neuron growth and electrophysiological recording.^6^ This result indicates that the nanocomposite has the potential to be used in biological applications related to electrical stimulation.

In Figure 7d and e, the GCD curves are shown with respect to different applied current densities for both the 12 and 15PPy@LCNF electrodes. The *C*_sp_ of the material was calculated from the discharge curve and is denoted beside the GCD curve as measured at a specific applied current density. At a charge/discharge current of 2 A g^–1^, their discharge curves are almost symmetrical to the corresponding charge curve without observing an obvious drop in the IR, representing characteristic capacitance behavior and sufficient conductivity. Here, *C*_sp_ is reported as 110 F g^–1^ and 139 F g^–1^ for 12 and 15PPy@LCNF, respectively. These values confirm that 12 and 15PPy@LCNF possess significant active surface areas available for efficient ion diffusion and transport. At 5 and 10 A g^–1^, an IR drop was observed in the GCD curves, suggesting that the IR of PPy@LCNF is limiting the charge–discharge process at these high charge current densities. This may reflect the intermediate level of protonation in the PPy@LCNFs. Still, the electroactive material of 12PPy@LCNF was able to retain 86% of its capacitance after 1200 GCD cycles at a charge/discharge current density of 5 A g^–1^. Compared with what was reported for the core–shell nanocomposite of PPy@CNC (∼300 F g^–1^), the capacitance values are lower.^27^ As smaller nanorods with regard to nanodimension in CNCs compared to CNFs, CNCs possess a larger gravimetric surface area to form an ultrathin conductive coating through the polymerization of pyrrole. Thus, less *C*_sp_ was anticipated for PPy@LCNFs than PPy@CNCs, providing that the ultrathin coating of PPy tightly conforms to the geometry of the nanocelluloses. Nevertheless, the utilization of LCNF as structural template has the advantage to result in nanostructured PPy in micrometer-scale geometry length, which eventually facilitates the distinct film- or foam-forming capacity of PPy@LCNFs, owing to the extensive fibril–fibril entanglement among the flexible submicrometer or several-micrometer-long fibrils.

PPy possesses excellent stimulus-responsive properties that make it a smart biomaterial, allowing for dynamic control of its properties by the application of an electric field.^9^ To reveal the electrochemical responsiveness of PPy@LCNFs, in situ UV–vis spectroscopy was deployed to detect the corresponding changes in the absorption spectra of PPy@LCNFs under an applied potential. The potential was applied in situ during the measurement of the UV–vis spectra and stepwise shifted with an interval of 0.1 V and progressively shifted from −0.5 to 0.5 V in 0.1 M KCl. Figure 7g and h show successive UV–vis spectra of the spray coating of 12 and 15PPy@LCNF on ITO glass, respectively. For 12PPy@LCNF, the most characteristic changes in the spectra were observed at λ = 394, 479, 519, and 700–1300 nm (Figure 7g), which are in good accordance with previous studies.^69^ The strong absorption band at 394 nm (3.15 eV) reveals an electron transition from the valence band to the conduction band and is assigned as the π–π* transition in the aromatic form of the neutral polymer. The other bands are assigned to transitions inside the band gap, i.e., transitions between energetic levels of polaron/bipolaron. The absorption peak at 479 nm (2.58 eV) is characteristic of an electron transition from the valence band to the antibonding polaron/bioplaron level. The absorption band at 519 nm (2.39 eV) might be associated with polarons, which exists in the spectra even under −0.5 V. The broad absorption band in the range from 700 to 1300 nm is due to electron transition from bonding to the antibonding polaron (radical cation)/bipolaron (dication) state. The reduced film (*E* = −0.5 V) had an absorption maximum at 394 nm, and this maximum shifted to higher energies upon oxidation and decreased in intensity successively. Simultaneously, the intensity of the absorption band at 479 nm increased and the intensity of the absorption band at 519 nm decreased, until the potential of the electrode reached +0.1 V. Further oxidation of the film caused a rapid increase in the intensity of the broad absorption band located between 700 and 1300 nm. This suggests that PPy@LCNFs are converted from the neutral to the cation radical form (conductive) upon the positive shift of the applied potential. It can also be seen that the π–π* transition band in the neutral form is at a wavelength in 12PPy@LCNF (394 nm) that is lower than that in 15PPy@LCNF (402 nm), indicating a larger band gap in the former. Meanwhile, the polaron band started to increase steeper in 12PPy@LCNF than in 15PPy@LCNF, which indicates that 12PPy@LCNF might be more susceptible to oxidation in converting from its neutral state to its conducting state. In the conducting state, the π–π* transition band of 12PPy@LCNF is also narrower than that in 15PPy@LCNF. This indicates that the molecular structure might be more ordered in the former case, which is in good accordance with the observed higher DC conductivity for 12PPy@LCNF than for 15PPy@LCNF.

### Cytotoxicity Evaluation of PPy@LCNFs

**Figure 8:**
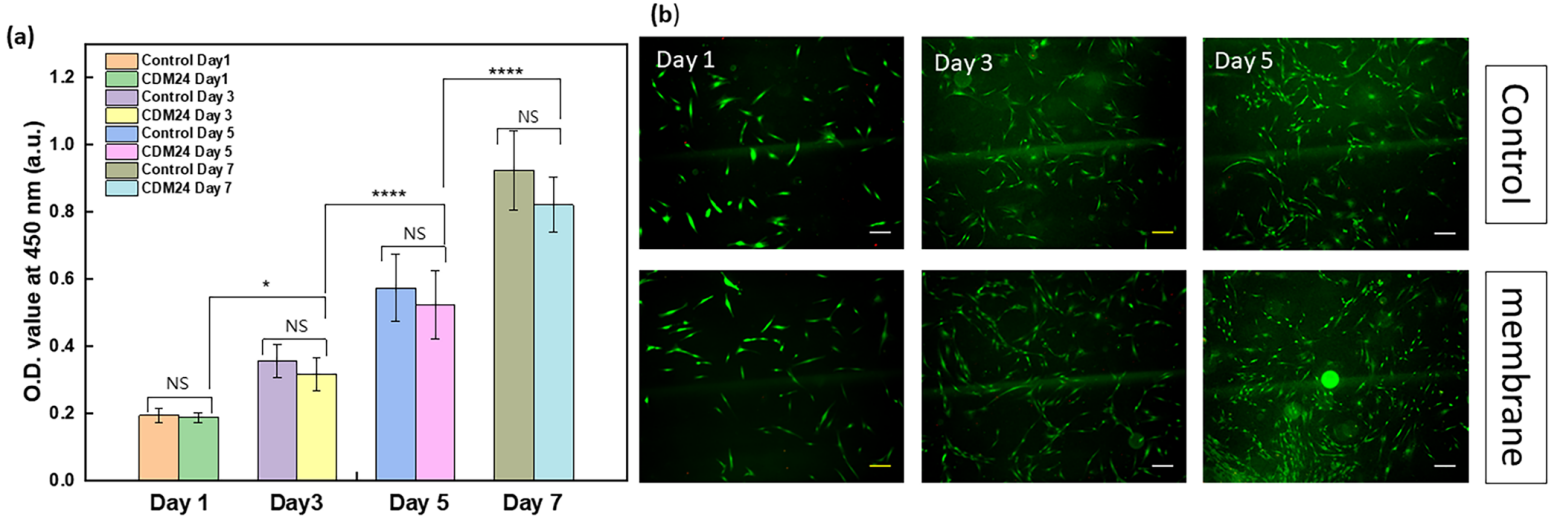
Cytotoxicity evaluation of the 12PPy@LCNF. (a) Optical density (O.D.) value of NHDFs from control and CDM 24 on Day 1, Day 3, Day 5, and Day 7 determined by CCK-8 assay (NS: not significant). (b) The fluorescence microscopy images of indirect-contacted NHDFs on Day 1, Day 3, and Day 5 (Live: green, Dead: red; Scale bars: 200 μm).

The presence of bipolarons in protonated PPy supports both mixed electronic and ionic conductivity. This type of material enables potential distribution in response to electrochemical or electrical stimuli, which is of great interest in the biomedical field. Previous studies have shown that PPy has the advantages of (i) adapting to a wide range of cell types (in vitro), (ii) no significant cytotoxicity, and (iii) stimulating neural regeneration in vivo.^70^ To establish a reference for using the nanocomposite PPy@LCNFs as a functional biomaterial in bioelectronics interfacing, the cytocompatibility must be evaluated. In this regard, we carried out extracts and indirect contact tests with NHDFs cell lines, in line with the standard of biological evaluation of medical devices (ISO 10993–5). The extract method verifies whether the material is harmful to biological cells by comparing the proliferation of NHDF cells in CDM24 and in the untreated medium (Control). Cell proliferation was quantified by CCK-8 in three independent experiments and the average was obtained (±SE). Significance analysis by GraphPad Prima 9 showed that there was no significant difference (NS) between the proliferation of NHDF cells in the CDM24 and in Control groups over 7 days. Cells in the Control and CDM24 groups both proliferated significantly for 7 days (Figure 8a and Figure S8). This confirms that the extract solutions did not have adverse effects on the cell cultures. The presence of membrane cytotoxicity was verified using the indirect contact method by analyzing Live/Dead fluorescence images of cells in two distinct groups. The experimental group consisted of cells with the insert and membranes (placed 0.8 mm above the cell layer), while the control group involved only the insert. This analysis was performed over a period of 5 days. The NHDF cell density and morphology were comparable between the control group and the membrane group (Figure 8b). This suggests that the membrane presence did not elicit any discernible impact on the inherent proliferation of NHDFs.

## Conclusions

When LCNFs are used as the structural template to modulate the morphology of PPy in chemical oxidation of pyrrole by Fe(III) ions, complexation of Fe(III) ions with aromatic lignin induces surface-confined polymerization of pyrrole on the nanofibrils. This synergetic strategy effectively yields 1D nanomaterial of PPy@LCNFs in a core–shell configuration with the PPy nanocoating tightly conforming to the morphology of cellulose nanofibrils, on a submicrometer and micrometer scale in terms of the fibril length. Multiple spectroscopic experiments confirmed the oxidation state of PPy in PPy@LCNFs as an intermediate protonation with respect to the imine-like N atoms. The highly positive surface charge distributed over the fibrils bestowed a durable aqueous dispersity to the PPy@LCNFs. Fibril–fibril entanglement that is inherited from the structural template provides enhanced mechanics and flexibility to the solid form of PPy@LCNFs enabling processing with versatile means. The hybrid nanostructured PPy with lignocellulose nanofibrils endows the solid-form PPy@LCNFs with a high electrical conductivity in the magnitude of 1–12 S cm^–1^, dependent on the network connectivity. PPy@LCNFs were also shown to be electroactive with superior capacitance and electrochemically stable. The doping/undoping of PPy@LCNFs was responsive to the applied electric field as a stimulus. Vacuum-filtered membrane of 12PPy@LCNF showed no apparent cytotoxicity in a noncontact cell culture of fibroblasts. All of these material properties underpin the promises for 12PPy@LCNF as a flexible conductive element in fabrication of flexible and metal-free electrodes interacting with biological systems, i.e., for neural interfacing and tissue engineering.

## Supporting Information Available

Synthesis protocol of nanocellulose dispersions; Shelf stability of dispersions of PPy@nanocelluloses in one month; TEM/SEM images of LCNF/PPy@LCNFs; Fe(III) leaching during the dialysis of 12PPy@LCNF when 0.3 M HCl was involved in the synthesis protocol; XPS survey analysis and the binding energy/area percent of the PPy@LCNFs; Gravimetric capacitance, 200 potential cycles and Nyquist plots of PPy@LCNFs; Protocols of cell maintenance, bioassays, and cell imaging; Fluorescence microscopy images of NHDFs adhered on the culture plate bottom. (PDF)
