# Supplementary material for: Aqueous Processable One-Dimensional Polypyrrole Nanostructured by Lignocellulose Nanofibril: A Conductive Interfacing Biomaterial
Source: Biomacromolecules. 2023 Jul 12;24(8):3819–34. doi: 10.1021/acs.biomac.3c00475 (PMC10428162; doi:10.1021/acs.biomac.3c00475)
Supplement: Supplementary file 1 — bm3c00475_si_001.pdf [file bm3c00475_si_001.pdf]

Aqueous processable one-dimensional polypyrrole nanostructured by lignocellulose nanofibril: A conductive interfacing biomaterial

Shujun Liang[a, c 1], *Wenyang Xu*[a,1], *Liqiu Hu*[a], *Ville Yrjänä*[b], *Qingbo Wang*[a], *Emil Rosqvist*[b], *Luyao Wang*[a], *Jouko Peltonen*[b], *Jessica M. Rosenholm*[c], *Chunlin Xu*[a], *Rose-Marie Latonen*[b], *Xiaoju Wang*[a, c*]

[a] Laboratory of Natural Materials Technology, Faculty of Science and Engineering, Åbo Akademi Un-versity, Henrikinkatu 2, Turku FI-20500, Finland

[b] Laboratory of Molecular Science and Engineering, Faculty of Science and Engineering, Åbo Akademi University, Henrikinkatu 2, Turku FI-20500, Finland

[c] Pharmaceutical Sciences Laboratory, Faculty of Science and Engineering, Åbo Akademi University, Tykistökatu 6A, Turku FI-20520, Finland

[1] S. Liang and W. Xu are equally contributing first-authors.

[*]Corresponding author: Xiaoju Wang and email address: xwang@abo.fi

Detailed protocol for the synthesis of lignocellulose nanofibril (LCNF) and TEMPO-oxidized cellulose nanofibril (TCNF)

The LCNF was prepared from the never-dried unbleached kraft birch pulp (2 wt%) with a lignin content 5~6%, through the grinding and homogenizing treatment. To obtain the gel-like fibril suspension, the pulp was sufficiently swollen in distilled water overnight and then mechanically defibrillated with a MKCA6-5J CE SuperMassColloider (Masuko Sangyo Co. Ltd, Saitama, Japan). The gap between the two grinding stones was adjusted gradually, at ~0 µm, ~50 µm, ~100 µm and ~150 µm at 800~1, 000 rpm for 1~2 cycles, respectively, followed by at ~200 µm at 1, 450 rpm for 3 cycles. The obtained suspension was further diluted to 1 wt% with distilled water and homogenized by a high-pressure homogenizer (AH-100D, ATS Engineering Co., Ltd., China) under 1,000 bar for 10 cycles. The obtained well-dispersed LCNF suspension was cleansed with Milli-Q water by centrifuging until the conductivity of the filtrate was close to the distilled water (~ 1.5µS cm^{-1}) and passed through the homogenizer under the same condition again.

The TCNF was prepared using bleached kraft birch pulp. 10 g of the fibre was disintegrated in 900 mL distilled water. The solution of TEMPO (0.1 mmol/g fibre) and sodium bromide (1.0 mmol/g fibre) were added to reach the fibre dispersion consistency of 0.1%. The oxidation of the slurry was initiated by the dropwise addition of NaClO (10 mmol/g fibre) solution (12 wt%. active chlorine) after the pH was adjusted to 10.0 by 0.5 M NaOH. Meanwhile, 0.5 M NaOH was used to maintain the pH of the system at 10.5 during the reaction period of 1.5 h. After the oxidation, the pH of the system was adjusted to 7.0 using 1.0 M HCl. The oxidized fibres were purified with deionized water by filtration until the conductivity of the filtrate was below 4.5 µS cm^{-1} and then homogenized under a pressure of 1,000 bar for 10 cycles to produce nanocellulose. The charge density (carboxylic acid content) of TEMPO-CNF was 1.66 ± 0.03 mmol/g as determined by potentiometric titration.

Shelf stability of dispersions of PPy@nanocelluloses in one month

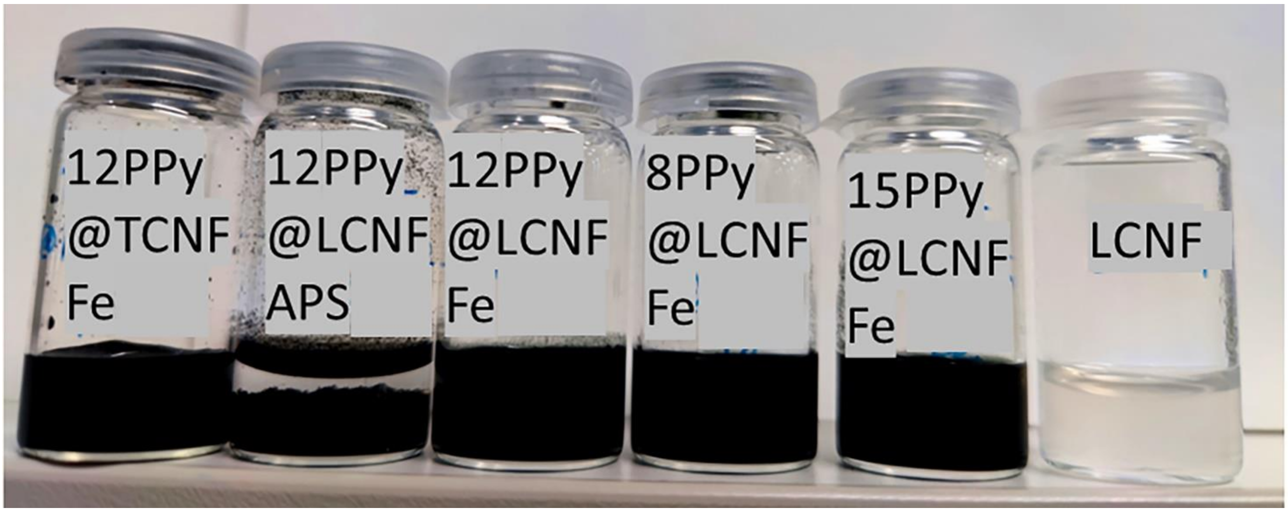

Figure S1. Images of PPy@TCNF and PPy@LCNFs dispersions (0.2 wt%, 2mL) after one-month storage at room temperature when using Fe(III) or ammonium persulfate (APS) as oxidant.

TEM/SEM images of PPy@LCNFs

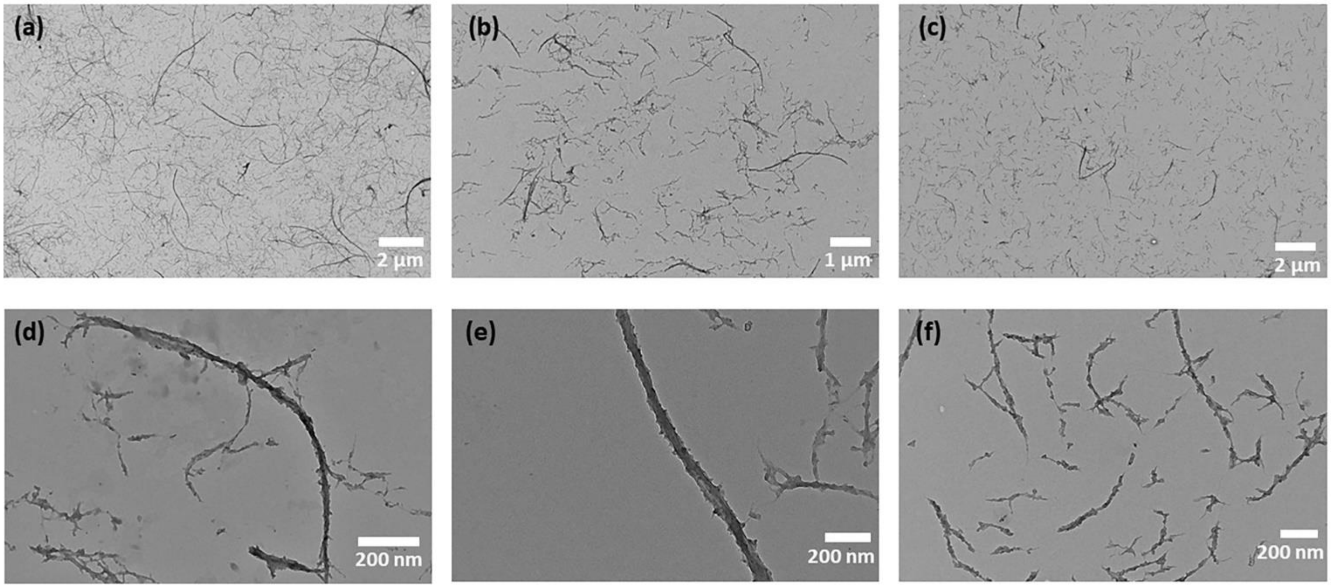

Figure S2. (a)-(c) TEM images in low magnification for overview of the nanofibril size distribution in PPy@LCNFs: (a) 8PPy@LCNF in 4, 000x; (b) 12PPy@LCNF in 5, 000x; and (c) 15PPy@LCNF in 5, 000x. (d)-(f) TEM images in high magnification for close visualization of the nanomorphology in PPy@LCNFs: (d-e)individual microfibril in 12PPy@LCNF in 15,000 and 30, 000x, respectively; (f) nanofibrils in 15PPy@LCNF in 25, 000x.

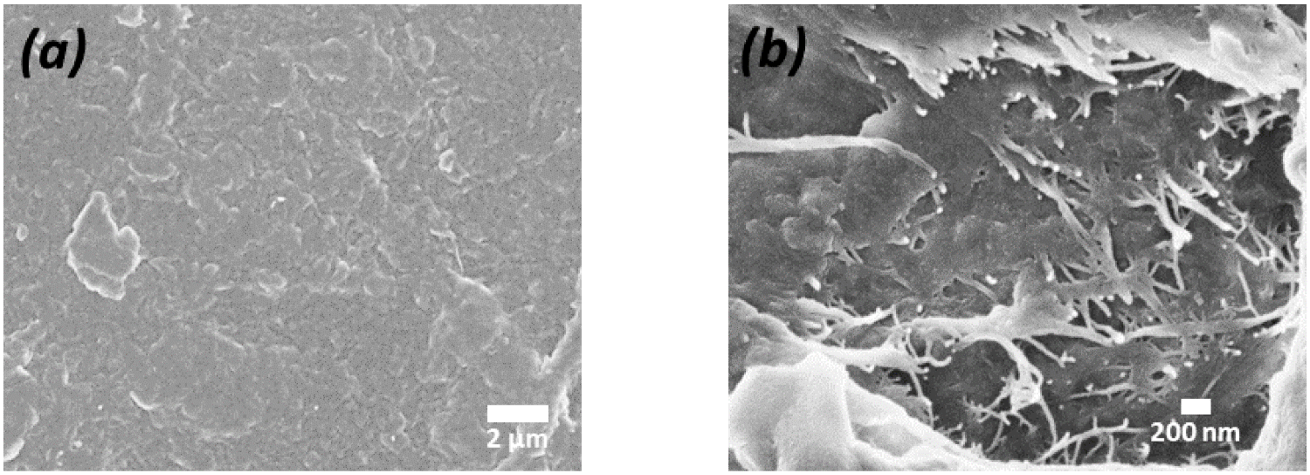

Figure S3a. Morphology of LCNF under SEM images with 5, 000 x(a) and 25, 000 x (b). Carbon is sputtered as a surface conductive coating for SEM observation.

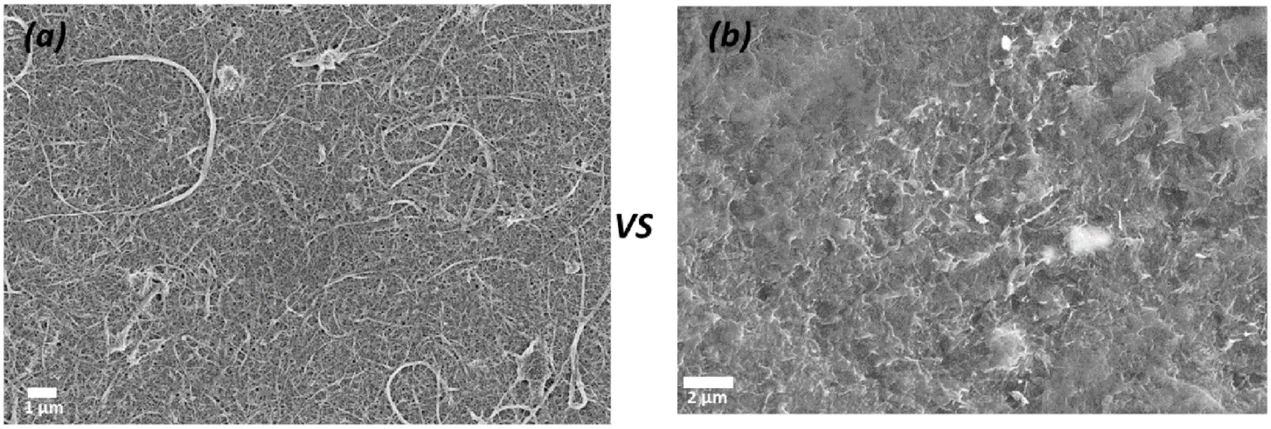

Figure S3b. Surface morphology of solid forms of 12PPy@LCNF under SEM imaging: (a) spray coating on glass and (b) vacuum-filtrated membrane. Magnification: 5, 000x for (a) and (b). No sputter coating is needed for SEM observation.

Fe(III) leaching during the dialysis of 12PPy@LCNF when 0.3 M HCl was involved in synthesis protocol.

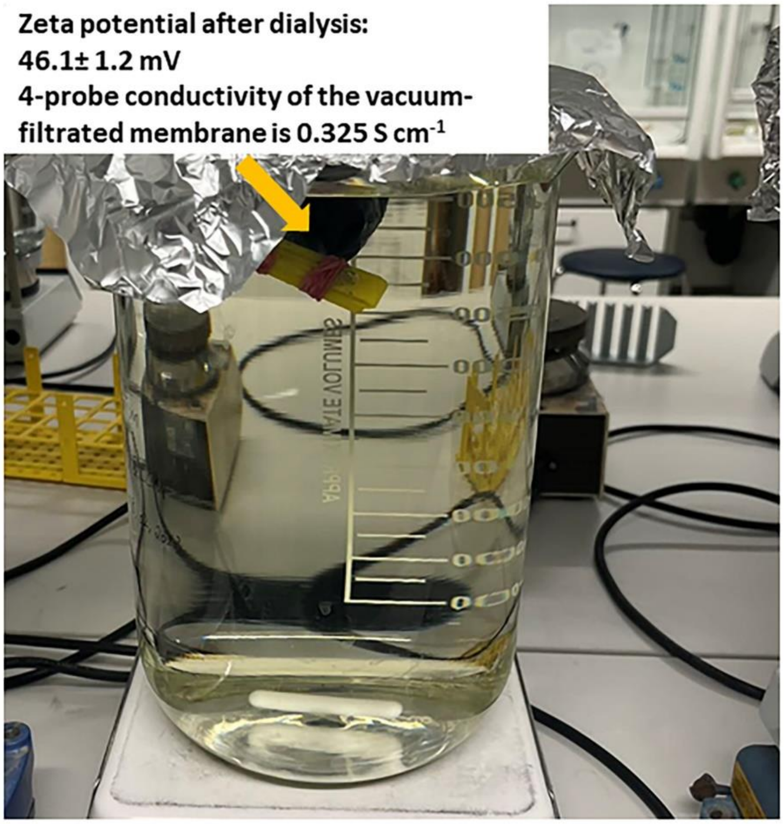

Figure S4. The colour change during the dialysis procedure indicated that Fe(III) ions leached out from 12PPy@LCNF/Fe(III) when 0.3 M HCl was involved in the synthesis protocol.

The protocols are described as below:

The 12PPy@LCNF (Fe/0.3 M HCl) dispersion was obtained with the same experimental conditions and processes as before (Seen *Section 2.1 Nanomaterials synthesis*). However, the ratio of LCNF to Milli-Q water was changed from 1:1 to 3:2, where 1:3 of Milli-Q water was used to prepare 0.3 M HCl. The diluted LCNF was mixed with pyrrole and stirred with 0.3M HCl for 1 h. The oxidant, $FeCl_3 \cdot 6H_2O$ dissolved in 4 mL Milli-Q water, was added and the synthesis was continued for 3 h. After dialysis of 12PPy@LCNF (Fe/0.3 M HCl), the conductivity of the vacuum-filtrated membrane was measured using the 4-probe conductivity method.

XPS survey analysis and the binding energy/area percent of the PPy@LCNFs

Table S1a. XPS survey analysis of PPy@LCNFs.

**Binding energy (eV)**	**Element**	**8PPy@LCNF (atomic%)**	**12PPy@LCNF (atomic%)**	**15PPy@LCNF (atomic%)**
286.42	C 1s	69.11	75.13	72.51
534.01	O 1s	20.91	16.17	14.36
401.45	N 1s	8.66	7.05	11.91
713.28	Fe 2p	0.64	0.30	0.30
199.78	Cl 2p	0.22	0.49	0.75
348.93	Ca 2p	0.46	0.86	0.16
	Cl/N	0.025	0.069	0.063

Table S1b. Binding energies and area percent of the deconvoluted bands in the N 1S core level region in the XPS spectra of PPy@LCNFs.

**core-level regions**	**bands**	**8PPy@LCNF**		**12PPy@LCNF**		**15PPy@LCNF**		**band assignment**
		BE (ev)	area (%)	BE (ev)	area (%)	BE (ev)	area (%)	
N 1s	N_1	398.27	13.7	398.24	12	397.73	13.9	Uncharged deprotonated imine N atoms (e.g., =N-) or due to imine defects responsible for interruption of effective conjugation
	N_2	400.10	80.6	400.10	80.0	399.68	77.5	Neutral N atoms (e.g.,-NH-) in the polymer
	N_3	402.21	5.6	402.16	8	401.80	8.5	Protonated imine N^+ (e.g., $=NH^+$-) or high oxidation states of the N atoms
	N_3/N_1	0.41		0.67		0.61		

Gravimetric capacitance, 200 potential cycles of CV, and Nyquist plots of PPy@LCNFs

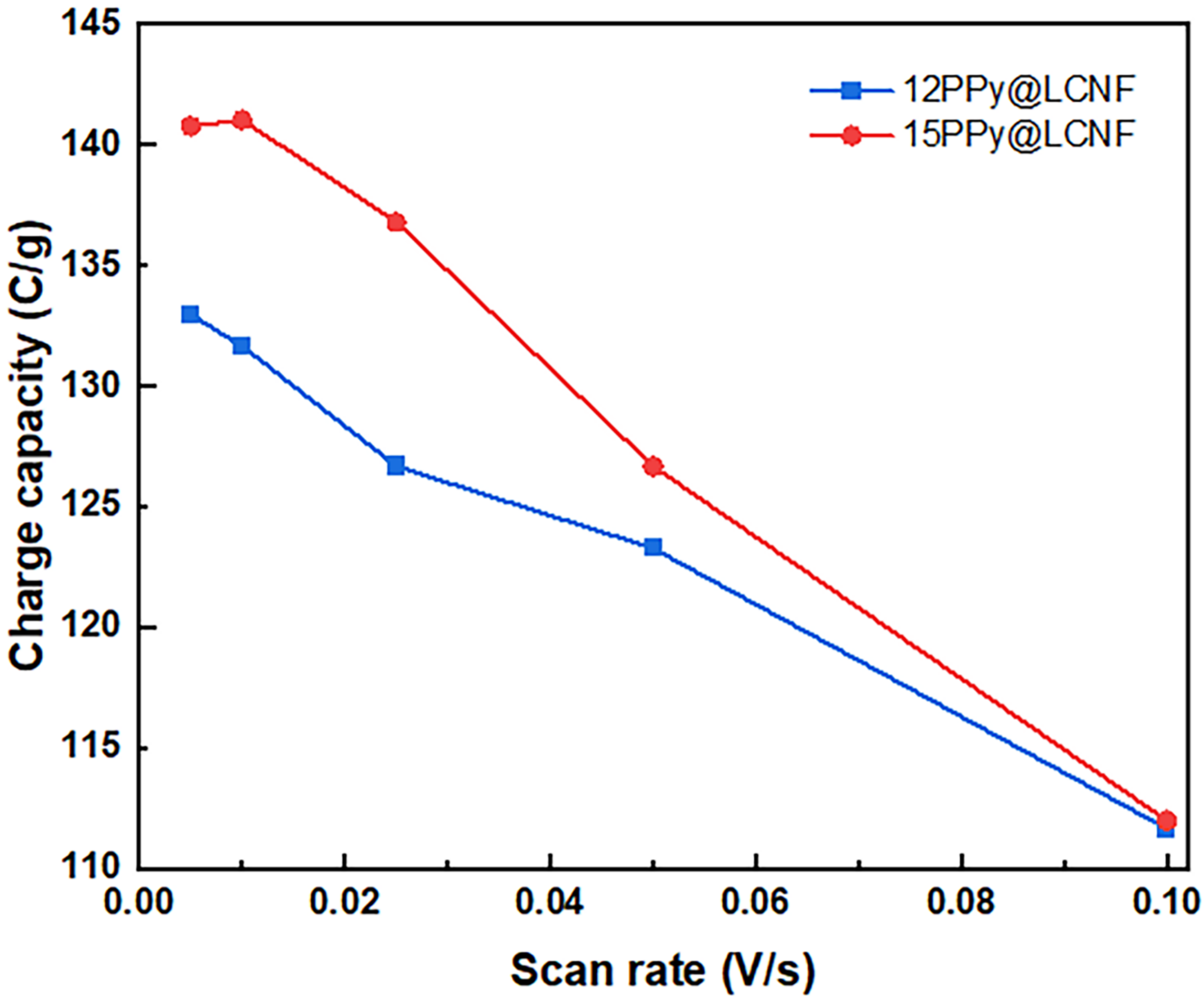

Figure S5. The gravimetric capacitance of 12PPy@LCNF as calculated from CVs with respect to scan rates increased from 0.005 to 0.1V s^{-1}.

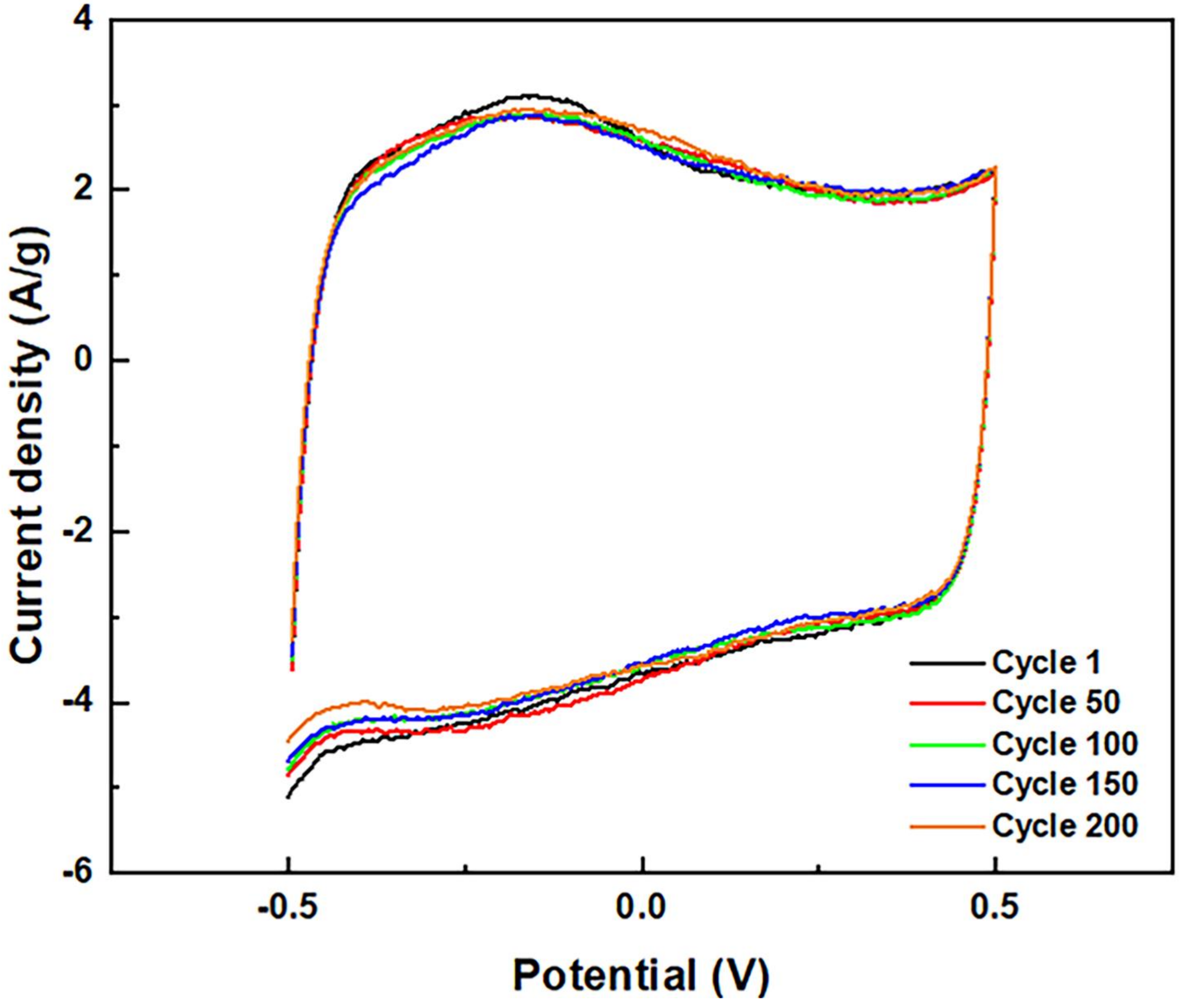

Figure S6. 200 potential cycles of 12PPy@LCNF with a scan rate at 0.025 V s^{-1}.

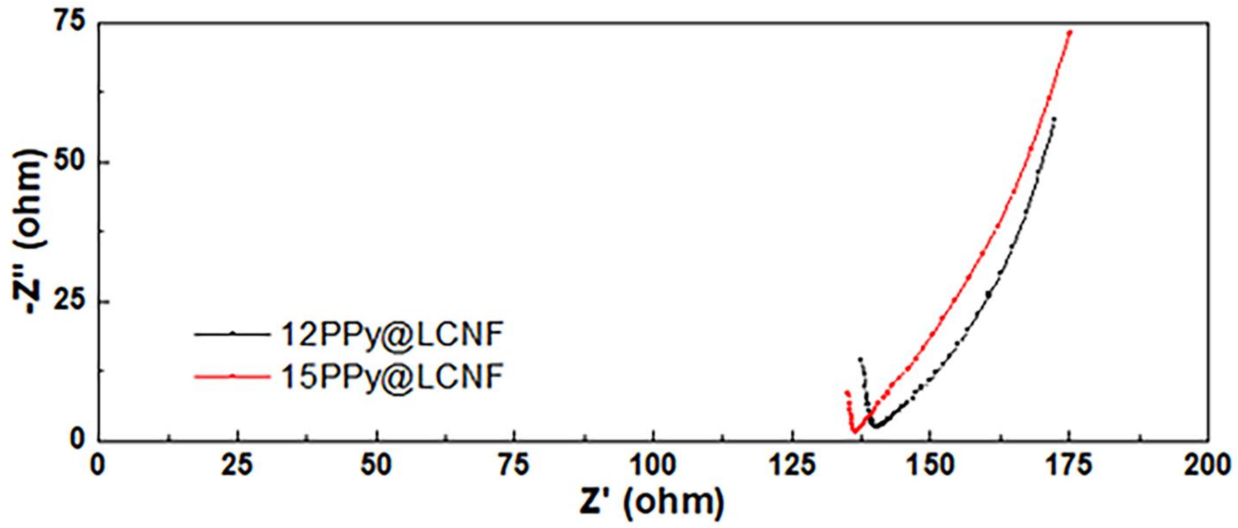

Figure S7. The Nyquist plots of 12 and 15PPy@LCNF as measured by electrochemical impedance spectroscopy (EIS) in 0.1 M KCl electrolyte solution.

Protocols of cell maintenance, bioassays, and cell imaging

The NHDFs were cultured in the complete cell culture medium and passaged about twice a week. The complete cell culture medium contained Dulbecco's Modified Eagle's Medium (DMEM) with fetal bovine serum (FBS, 10%) and penicillin streptomycin (Pen-strep, 100 U mL^{-1}) and replaced every second day. The NHDFs were cultured in an incubator with a 5% CO_2 atmosphere at a constant temperature of 37 °C and kept before passage 12.

CCK-8 and Live/Dead assay: The CCK-8 testing reagent was prepared by diluting the CCK-8 agent (CK04-11, Dojindo) with complete cell culture medium (v:v, 1:10). At specific time intervals, the cell culture medium in each well was substituted with 150 μL CCK-8 testing reagent then cultured for 1 hour in the incubator. After that, the 110 μL CCK-8 testing reagent was absorbed to a new plate and reading by a spectral scanning multimode reader (Varioskan Flash, Thermo Scientific) at 450 nm absorbance. The cells viability was evaluated by the Live/Dead assay. The culture medium was discarded and the cells were stained by the 200 μL staining reagent for 1h after washing with PBS. The staining reagent was prepared by mixing 0.5 μM Calcein-AM/1.6 μM EthD-III in PBS. The fluorescence microscopy images were obtained with the Ziess Axio Vert.A1 and further processed using Fiji ImageJ software.

Fluorescence microscopy images of NHDFs adhered on the culture plate bottom in extracts test

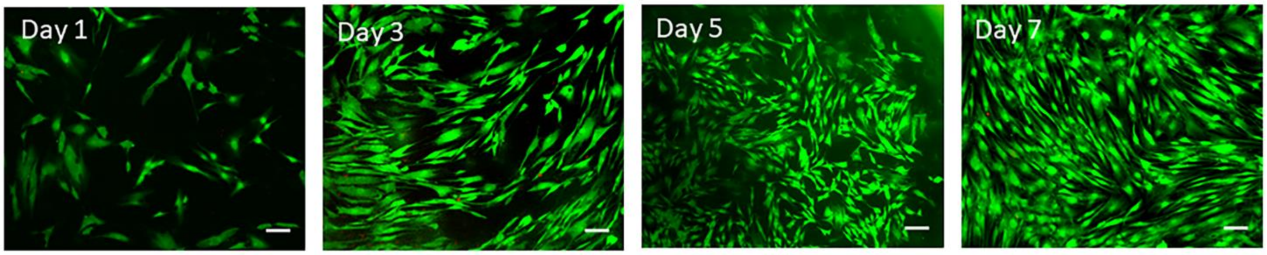

Figure S8. The fluorescence microscopy images of NHDFs from CDM 24 on Day 1, Day 3, Day 5 (Live: green, Dead: red; Scale bars: 100 μm).
